# Nutrition and growth of primary ciliary dyskinesia patients: a systematic review

**DOI:** 10.1183/16000617.0024-2026

**Published:** 2026-05-27

**Authors:** Nena Karavasiloglou, Vasiliki Gkatzou, Andrea Fernandez-Rodriguez, Valérie Schwartz, Myrofora Goutaki

**Affiliations:** 1 Institute of Social and Preventive Medicine (ISPM), University of Bern, Bern, Switzerland; 2 Graduate School for Health Sciences, University of Bern, Bern, Switzerland; 3 Division of Paediatric Respiratory Medicine and Allergology, Department of Paediatrics, Inselspital, Bern University Hospital, University of Bern, Bern, Switzerland

## Abstract

**Background:**

Growth, nutritional status and intake can be crucial for the management and prognosis of chronic lung diseases, including primary ciliary dyskinesia (PCD); yet research in PCD is scarce. In this study, we systematically reviewed the existing literature and summarised the evidence on growth, nutritional status and intake of patients with PCD.

**Methods:**

We conducted searches in Medline, Embase, Scopus and PubMed limited to the past 20 years, due to changes in the diagnosis of PCD. Eligible studies included cross-sectional, case–control and prospective studies, and case series with >5 people that reported on the growth, nutritional status or intake of patients with PCD. We performed additional searches through reference list checking and in medRxiv for relevant preprints. The review was registered in PROSPERO (CRD420251065699).

**Results:**

50 studies were included in the qualitative synthesis. We observed variability in growth-related outcomes, with some studies reporting reduced z-score for height, body weight or BMI, while others reported no growth differences in patients with PCD. The prevalence of undernutrition ranged from 4 to 11%; underweight ranged from 0 to 50%. Overweight and obesity estimates ranged from 3 to 25%. The prevalence of vitamin D insufficiency was 54%, while deficiency was reported in 18–26% of study participants. Only three studies reported on the nutritional intake of patients with PCD.

**Discussion:**

We observed considerable variability in growth-related outcomes and a lack of nutritional intake-related data in patients with PCD. The findings we noted related to malnutrition and vitamin D insufficiency/deficiency are alarming and warrant further investigation.

## Shareable abstract

**The findings of this first systematic review of growth and nutrition in primary ciliary dyskinesia (PCD) highlight the need for timely and frequent assessment of nutrition and growth as standards of care in PCD.** https://bit.ly/41jHpnv

## Graphical abstract

**Figure:**
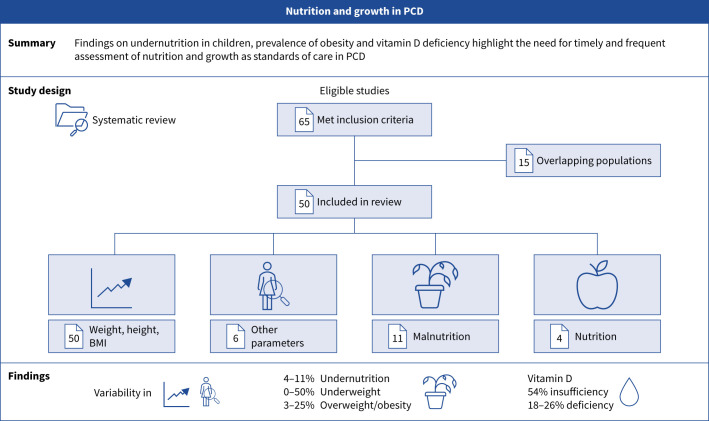
This systematic review reports on growth and nutrition-related outcomes in people with primary ciliary dyskinesia (PCD) and highlights the need for their timely and frequent assessment as standards of care. BMI: body mass index.

## Introduction

The relationship between growth, nutrition and respiratory diseases is multifaceted and potentially bidirectional. On the one hand, chronic respiratory diseases or their treatment could lead to delayed growth [1, 2]. Furthermore, frequent respiratory infections can lead to a higher caloric need due to increased metabolic demands and reduced appetite. In contrast, growth and good nutritional status have been linked to improved lung function [3–5]. Possibly due to differences in disease aetiology or lack of extensive research on the topic, there is no scientific consensus on the optimal nutrition for respiratory health. So far, most studies have focused on common respiratory conditions like asthma [3, 6] or diseases where nutrition is clearly affected and thus closely monitored, such as cystic fibrosis (CF) [5, 7]. Studies in other conditions are limited and little is known about the growth and nutritional intake in less common chronic respiratory diseases.

Primary ciliary dyskinesia (PCD) is a rare, genetic, multiorgan disease that affects around 1 in 7500 people [8]. PCD is characterised by dysfunctional cilia and impaired mucociliary airway clearance leading to chronic rhinosinusitis, bronchiectasis and hearing impairment. Manifestations from other organ systems include situs abnormalities, congenital heart defects and fertility issues [9–11]. Due to the impaired mucociliary airway clearance, patients frequently experience recurrent respiratory exacerbations and require continuous treatment and physiotherapy. Despite the above-mentioned relationship among growth, nutrition and respiratory disease, few studies have investigated growth or nutrition in patients with PCD, mainly as single centre cross-sectional analyses of available hospital data. Furthermore, the evidence is mostly reported as secondary end-points in studies, not allowing to evaluate proper assessment.

In this systematic review, we aimed to identify for the first time studies reporting on growth and nutrition in patients with PCD and summarise the available evidence across different growth- and nutrition-related parameters.

## Methods

### Search strategy and selection criteria

The review protocol has been published in PROSPERO (under CRD420251065699) and our reporting adheres to the Preferred Reporting Items for Systematic Reviews and Meta-analyses (PRISMA) guidelines [12].

We conducted literature searches in Medline (Ovid), Embase (Elsevier), Scopus (Elsevier), and PubMed (NLM) on 12 June 2025 using Medical Subject Heading (MeSH) terms. We set a 20-year time limit (articles published after 1 January 2005), considering changes in the diagnosis of PCD during the past 20 years. The full search strategies are available in the supplementary methods. We checked medRxiv for potential preprints relevant to our research question on 5 August 2025. We additionally employed reference list checking techniques.

We defined our eligibility criteria as human observational cross-sectional, case–control and prospective studies, or case series with more than five patients, reporting on the growth, nutritional status or nutritional intake in patients with PCD. For growth, we considered studies including height or length, weight, body mass index (BMI) or other growth-related measurements (*e.g.* body composition). We excluded studies on populations with other diagnoses, studies not reporting on the nutritional status, nutritional intake or growth, as well as studies of all other study designs, including reviews, conference abstracts and opinion pieces. We applied no restrictions regarding participant age, study duration or language. Two reviewers screened the retrieved articles independently at the title and abstract and the full-text level against the eligibility criteria using Covidence (Veritas Health Innovation, Melbourne, Australia, www.covidence.org). Reviewers remained blinded until the screening was completed at each level. We resolved any disagreements after discussion with a third experienced reviewer (M. Goutaki).

### Data extraction

After the full-text screening, we extracted data from the included publications in a standardised form. The extracted data included basic information on the publication, the study population and on the parameter(s) of interest (height or length, weight and BMI; other growth parameters; nutrition), their assessment method, and relevant findings. We only considered multiple publications from the same study if they significantly differed from each other, either in the parameter assessed, the type of analysis (cross-sectional *versus* longitudinal) or the time period of data collection, to ensure minimal overlap in included populations and data.

Due to heterogeneity in the reported parameters and their definitions, we did not perform a meta-analysis. Instead, we qualitatively synthesised the results and grouped and presented them by parameters of interest, namely height or length, weight, and BMI, other growth parameters, and nutrition.

### Risk of bias assessment

We assessed the risk of bias of included studies using a modified version of the Agency for Healthcare Research and Quality tool, as cited in Mamikutty *et al*. [13]. We adapted the questions and the scoring approach, as necessary, and developed detailed instructions for assessors to streamline the assessment (supplementary methods). Two assessors assessed and scored the articles independently, following the instructions mentioned above. Assessors remained blinded until the scoring was completed. The assessors discussed their scorings, a third experienced assessor (M. Goutaki) resolved disagreements in scoring when necessary, and assessors came to a consensus for the final scoring of each item for the included studies. We calculated article quality based on the sum of high-scoring items (*i.e.* those scored with 1) and classified into three groups: low quality=0–2; moderate quality=3–5; and high quality=6–7.

## Results

**FIGURE 1:**
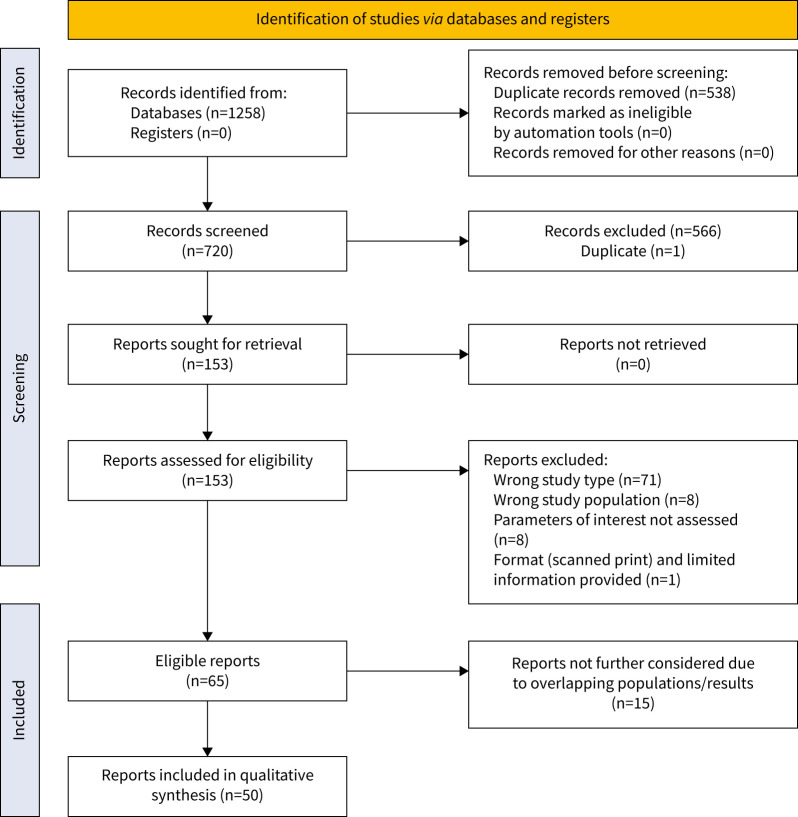
PRISMA (Preferred Reporting Items for Systematic Reviews and Meta-Analyses) flow diagram for study selection.

Our search identified 1258 records. After duplicate removal using the Deduklick tool [14], we assessed 720 records at the title and abstract level, 153 at the full-text level, and we identified 65 as eligible for inclusion (figure 1). We did not identify any preprints or additional references relevant to our research question through the additional searches mentioned in the Methods section. Due to considerable overlap in the eligible studies, we retained only 50 publications [15–64]. The selection was based on the time period of data collection (when available), the type of analysis (cross-sectional *versus* longitudinal) and the relevant parameters reported in each publication. Exceptionally, we included information on handgrip strength from an additional publication [65], since this data was not included in the larger study we selected from this population [34]. As such, we did not count this additional publication in the total count of retained publications and we did not assess its risk of bias.

Even though studies were conducted worldwide, most of the results came from Europe and North America. The included studies varied considerably in study size, from six to 1609 participants. Four included only adults, 22 included only children, and the remaining 24 studies included both paediatric and adult populations.

All identified studies reported on growth-related outcomes, *i.e.* height or length, weight, or BMI. Additionally, six of them also reported on other growth-related parameters and four on the nutrition-related parameters of patients with PCD.

Overall, we judged most studies to be at moderate risk of bias (42, 84%; score 3–5; supplementary table S1). We judged three of the studies (6%) as low risk of bias (score 6). No study achieved a score of 7. We judged five studies (10%) as high risk of bias (score 0–2). The results of our risk of bias assessment should be interpreted with caution since in most studies, growth, nutritional status or nutritional intake of patients with PCD were secondary end-points and their assessment was not reported in the publications with the necessary level of detail; therefore, these results do not reflect on the overall quality of these studies.

### Height (or length), weight and BMI

We noted considerable variability in reported height or length, weight and BMI (supplementary table S2). Some studies reported reduced z-score for height, body weight or BMI, while others reported no growth differences in patients with PCD, compared to the population reference standards. Furthermore, studies reported differences either by age (adult *versus* paediatric populations), across ciliary ultrastructural defect or genetic pathogenic variant groups or based on specific characteristics (*e.g.* under treatment, with lobectomy, *etc*.). The population reference standards used in the studies differed, with some using country-specific estimates and others comparing growth findings to international ones, such as those by the World Health Organization or the Centers for Disease Control and Prevention. Furthermore, many studies did not report the reference standard they used to calculate z-scores. This lack of reporting and use of a common population reference standard makes comparison across studies challenging. Other studies did not calculate z-scores at all and some reported the weight and height of mixed populations (*i.e.* adult and paediatric) together, which hinders our ability to draw overall conclusions for the height or length, weight, and BMI of patients with PCD across age groups and geographic locations.

Five studies reported longitudinally on the height or length [17, 22, 44, 52], weight [22, 44, 52] and BMI [17, 22, 25, 44, 52] of patients with PCD (table 1 and supplementary table S2). Varying results were reported in four studies regarding height, with one showing that most patients did not have impaired height at first measurement and over the study period [22]. Another study showed a greater increase in height percentile between two time-points in children treated with azithromycin compared to those who were not [44]. The third study reported a decline in height until the age of 9 years old, which then stabilised [17], and in the fourth, height percentile was reported to be higher in patients with PCD compared to patients with CF after 6 years of age [52]. Regarding weight, one of the three studies reported stable weight z-scores over the study period [22], the other found comparable changes between two time-points in children treated with azithromycin compared to those who were not [44] and the final study reported higher weight percentile in PCD compared to CF after 6 years of age [52]. When considering BMI, of the five identified studies, three reported BMI comparable to population reference standards and stable over the study period [17, 22, 25]. Children treated with azithromycin had comparable BMI changes over the study period compared to those who were not [44], and in the last study, BMI percentile was similar in patients with PCD compared to patients with CF until age 10; after that age, the BMI percentile was significantly lower in CF [52]. Comparisons across studies are challenging due to differences in the timing of assessments, the reporting (*e.g.* z-scores, percentiles), the number of assessments and the specificities of the included populations (*e.g.* grouped according to lung function trajectories or azithromycin treatment).

**TABLE 1:** Characteristics and findings of included studies reporting longitudinal results on the height or length, weight, and body mass index (BMI) in patients with primary ciliary dyskinesia (PCD)

First author, study name [ref.], year, country	Basis for PCD diagnosis	Study participants (n)	Age^#^	Sex (% female)	Parameter assessment	Growth comparator	Findings
**Svobodová [17], 2013, CZ**	The diagnostic criteria were 1) clinical findings consistent with a diagnosis of PCD (otosinopulmonary symptoms), 2) abnormal video/HSVM of cilia confirmed at least twice with an interval between examinations of at least 4–6 weeks, 3) TEM findings consistent with a ciliary movement abnormality	29 in total, 12–25, depending on the timing of assessment	14.5 (1.2–24) (median (range))	55, total study population	Body height was measured using a wall-mounted stadiometer according to routine medical practice and body length in children aged 1 year old was measured using a bodymeter	CZ data	Length/height, mean SDS±sem, 1 year: 0.40±0.24 Length/height, mean SDS±sem, 3 years: 0.16±0.23 Length/height, mean SDS±sem, 5 years: −0.13±0.21 Length/height, mean SDS±sem, 7 years: −0.54±0.19 Length/height, mean SDS±sem, 9 years: −0.67±0.21 Length/height, mean SDS±sem, 11 years: −0.52±0.24 Length/height, mean SDS±sem, 13 years: −0.53±0.23 Length/height, mean SDS±sem, 15 years: −0.53±0.29 Length/height, mean SDS±sem, 17 years: −0.67±0.38 BMI, mean SDS±sem, 1 year: −0.29±0.25 BMI, mean SDS±sem, 3 years: −0.42±0.33 BMI, mean SDS±sem, 5 years: −0.02±0.24 BMI, mean SDS±sem, 7 years: −0.26±0.20 BMI, mean SDS±sem, 9 years: −0.30±0.18 BMI, mean SDS±sem, 11 years: −0.10±0.21 BMI, mean SDS±sem, 13 years: −0.25±0.16 BMI, mean SDS±sem, 15 years: −0.21±0.29 BMI, mean SDS±sem, 17 years: −0.38±0.23
**Maglione [22], 2014, International**	Confirmed diagnosis according to 2009 ERS consensus statement	78–158, depending on the length of follow-up	Age at first spirometry: 8.7 (4.2–17.4) (median (range))	49	Measured, NOS	UK data	Height, median (range), cm, first measurement: 128.9 (99.5–184) Height, mean±sd, z-score, first measurement: −0.05±1.11 Height, median (range), cm, year 2: 137.3 (111–186) Height, mean±sd, z-score, year 2: −0.06±1.11 Height, median (range), cm, year 4: 148.8 (123–194) Height, mean±sd, z-score, year 4: 0.10±1.11 Height, median (range), cm, year 6: 159.8 (132–195) Height, mean±sd, z-score, year 6: 0.13±1.01 Weight, median (range), kg, first measurement: 27 (13.5–100) Weight, mean±sd, z-score, 1st measurement: 0.01±1.19 Weight, median (range), kg, year 2: 33.7 (18.7–103) Weight, mean±sd, z-score, year 2: 0.06±1.23 Weight, median (range), kg, year 4: 41.5 (23–103) Weight, mean±sd, z-score, year 4: 0.24±1.14 Weight, median (range), kg, year 6: 50.5 (29.9–114) Weight, mean±sd, z-score, year 6: 0.32±1.08 BMI, median (range), kg·m^−2^, first measurement: 16.5 (11.5–32.6) BMI, mean±sd, z-score, first measurement: 0.03±1.27 BMI, median (range), kg·m^−2^, year 2: 17.3 (11.6–33.3) BMI, mean±sd, z-score, year 2: 0.12±1.31 BMI, median (range), kg·m^−2^, year 4: 18.5 (13.4–33.6) BMI, mean±sd, z-score, year 4: 0.25±1.22 BMI, median (range), kg·m^−2^, year 6: 19.5 (14.1–37.2) BMI, mean±sd, z-score, year 6: 0.29±1.23 BMI <1.96, n (%), first measurement: 11 (7%) BMI >1.96, n (%), first measurement: 10 (6%)
**Guan [44], 2022, CN**	Ultrastructural defect or biallelic pathogenic variant; or 2/4 clinical features: unexplained neonatal respiratory distress, year-round daily cough or nasal congestion beginning before 6 months, organ laterality defect and nNO level, or Kartagener syndrome	71	Age at diagnosis: 7.6±3.3 (mean±sd)	Discrepant information given in the manuscript Either 45 or 39, depending on the information used	Collected *via* electronic medical records	NA	AZM-treated Height, mean±sd, percentile, baseline: 45.9±27.9 Changes of height, median (25–75), percentile, follow-up: 9.6 (−0.2–22.0) Changes of height, n (%), percentile, follow-up: Increased: 18 (75) Decreased: 6 (25) Weight, mean±sd, percentile, baseline: 33.1±31.0 Changes of weight, median (25–75), percentile, follow-up: 5.1 (−0.0–22.0) BMI, mean±sd, percentile, baseline: 20.7±28.6 Changes of BMI, median (25–75), percentile, follow-up: 1.5 (1.0–2.0) BMI, mean±sd, z-score, baseline: −1.67±1.82 Changes of BMI, mean±sd, z-score, follow-up: 0.44±1.0 AZM-untreated Height, mean±sd, percentile, baseline: 55.7±28.6 Changes of height, median (25–75), percentile, follow-up: 6.9 (−17.8–24.5) Changes of height, n (%), percentile, follow-up: Increased: 14 (64) Decreased: 8 (36) Weight, mean±sd, percentile, baseline: 36.8±26.9 Changes of weight, median (25–75), percentile, follow-up: 10.2 (−3.0–28.2) BMI, mean±sd, percentile, baseline: 24.2±26.3 Changes of BMI, median (25–75), percentile, follow-up: 1.7 (−6.4–19.7) BMI, mean±sd, z-score, baseline: −1.14±1.18 Changes of BMI, mean±sd, z-score, follow-up: 0.67±1.4
**Halbeisen, iPCD [25], 2022, International**	Distinguished three levels of diagnostic certainty: patients with definite PCD according to the ERS guidelines with a hallmark ultrastructural defect identified by TEM or pathogenic biallelic PCD genetic mutations; patients with probable PCD who had abnormal HSVM findings or low nNO; and patients diagnosed on clinical grounds with an incomplete diagnostic algorithm	486	Age at diagnosis: 8.64±6.1 (mean±sd)	49	NA	WHO data	BMI, mean±sd, z-score, first measure: −0.04±1.27 BMI, mean±sd, z-score, last measure: 0.01±1.21
**Kinghorn,** **GDMCC [52], 2024, International**	Abnormal ciliary ultrastructure by TEM and/or identification of two pathogenic variants in a PCD-associated gene, together with compatible clinical features	136	8.4±4.6 (mean±sd)	51	Collected at annual study visits	CDC data	Height, estimated mean (95% CI), percentile, age 2: 46.8 (39.4–54.2) Height, estimated mean (95% CI), percentile, age 6: 45.9 (41.7–50.1) Height, estimated mean (95% CI), percentile, age 10: 45.1 (41.2–49.0) Height, estimated mean (95% CI), percentile, age 14: 47.0 (43.1–50.9) Weight, estimated mean (95% CI), percentile, age 2: 50.6 (44.0–57.2) Weight, estimated mean (95% CI), percentile, age 6: 50.9 (47.2–54.7) Weight, estimated mean (95% CI), percentile, age 10: 51.3 (47.6–54.9) Weight, estimated mean (95% CI), percentile, age 14: 52.8 (49.1–56.4) BMI, estimated mean (95% CI), percentile, age 2: 55.0 (48.1–61.8) BMI, estimated mean (95% CI), percentile, age 6: 55.7 (52.0–59.4) BMI, estimated mean (95% CI), percentile, age 10: 56.3 (52.8–59.8) BMI, estimated mean (95% CI), percentile, age 14: 54.5 (51.0–57.9)

AZM: azithromycin; CDC: Centers for Disease Control and Prevention; CN: China; CZ: Czechia; ERS: European Respiratory Society; GDMCC: Genetic Disorders of Mucociliary Clearance Consortium; HSVM: high-speed video microscopy; iPCD: International PCD cohort; nNO: nasal nitric oxide; NA: not available; NOS: not otherwise specified; SDS: standard deviation score; TEM: transmission electron microscopy; UK: United Kingdom; WHO: World Health Organization. ^#^: Current age is reported, unless otherwise specified. Age reported in years, unless otherwise specified.

### Malnutrition

The prevalence of malnutrition, which encompasses both undernutrition and overweight/obesity, varied considerably across studies.

Six studies reported on undernutrition in children [22, 29, 32, 42, 43, 60]. Undernutrition was defined in studies as BMI z-score ≤−2 or BMI z-score ≤−1.96, while a study from the UK used an additional definition of height-for-age z-score ≤−2 [32]. The prevalence of undernutrition in children ranged from 4 to 11% based on the BMI z-score definition. When using the height-for-age definition, the prevalence of undernutrition was reported at 4.6%. While most studies pooled all children together due to small study population sizes, one study reported separately on undernutrition in children aged 0–6 years, which was reported at 2% [29].

Seven studies reported on underweight [26, 29, 40, 42, 45, 54, 63] and four on overweight [29, 42, 45, 63]. The prevalence of underweight ranged from 0% [45] to 50% [63]. The prevalence of overweight ranged from 3% [45] to 14% [29, 42]. Five studies reported on obesity [22, 26, 29, 42, 54], with the reported prevalence ranging from 4% [42] to 25% [26]. Finally, one study reported on the combined overweight/obesity estimate, based on a classification of BMI ≥25.0 kg·m^−2^, at 16.1% [40]. Of note, not all studies reported on how they defined underweight, overweight and obesity, which makes comparisons challenging.

### Other growth parameters

Only few studies reported on other growth-related parameters and always in conjunction with height, weight or BMI. The parameters investigated were body composition/lean body mass (n=2) [15, 32], handgrip strength (n=3) [39, 43, 65], muscle strength (n=2) [43, 60], triceps skinfold thickness (n=1) [39], mid upper arm circumference (n=2) [32, 39] and mid arm muscle circumference (n=1) [39] (table 2). Depending on the parameter, patients with PCD had comparable or slightly lower results than the comparator population. At times, the comparator was the expected reference values of the general population or children who are critically ill or, in some cases, patients with other respiratory diseases, such as CF.

**TABLE 2:** Characteristics and findings of included studies reporting on growth parameters other than height or length, weight, and body mass index (BMI) in patients with primary ciliary dyskinesia (PCD)

First author, study name [ref.], year, country	Basis for PCD diagnosis	Study participants (n)	Age^#^	Sex (% female)	Growth parameter	Growth parameter assessment	Findings
**Wells [15], 2011, CA**	NA	10	13.8±2.3 (mean±sd)	40	Lean body mass	MRI	Lean body mass, mean±sd, kg: 36.5±10.2
**Marino [32], 2019, UK**	Confirmed according to the ERS consensus guidelines	43 in total, 33–36 for our parameters of interest	7.0±3.6 (mean±sd)	49	Body composition	Bioelectrical impedance spectroscopy measurements were made using ImpediMedSFB7 (Pinkenba, QLD 4008 Australia) The machine was calibrated before use with a circuit of known impedance provided by the manufacturer Measurements were completed in unfasted subjects, were taken in triplicate and the mean used	MUAC, mean±sd, cm: 19.7±4.7 Fat mass mean±sd, kg: 5.9±6.7 Fat mass, mean±sd, %: 19.2±20.7 Fat-free mass, mean±sd, kg: 22.8±13.6 Fat-free mass, mean±sd, %: 80.7±79.2
**King [39], 2021, UK**	NA	25	23.0 (19.0–27.0) (median (IQR))	68	HGS, TSF, MUAC, MAMC	HGS was evaluated using a Takei 5401 Handgrip dynamometer (Takei Scientific Instruments Co., Ltd, Tokyo, Japan) This was performed with the participants in standing position, arm by their side with full elbow extension Measurements were repeated three times for the nondominant side Values were expressed as a mean of all three measures TSF was measured using Harpenden skinfold callipers (Baty International, Burgess Hill, West Sussex, UK) The mid-point was determined from the acromion to the olecranon process, and a skinfold measure was taken at the mid-point, with a mean determined from three repeated measures MUAC was recorded at this mid-point using a tape measure MAMC, was calculated from MAC and TSF using a standard formula: MAMC=MAC – (3.1415 TSF)	HGS, median (IQR), KgF: 15.6 (12.7–19.9) HGS, median (IQR), % norm: 58.0 (43.5–70.0) TSF, median (IQR), mm: 15.7 (12.4–18.1) TSF >50th percentile, n (%): 7 (28) MUAC, median (IQR): 29.0 (26.6–31.0) MAMC, median (IQR), cm: 23.6 (21.5–26.2) MAMC >50th percentile, n (%): 12 (48)
**Firat [43], 2022, TR**	HSVM or TEM or genetic testing in addition to typical clinical symptoms	27	10.74±4.01 (mean±sd)	59	Quadriceps femoris muscle strength Shoulder abductors muscle strength Elbow flexors muscle strength HGS	Muscle strength (quadriceps femoris, shoulder abductor and elbow flexor) was measured using a hand dynamometer (JTECH Power Track Commander, Baltimore, USA) HGS was measured using a grip dynamometer (Jamar, Fabrication Enterprised Inc, Irvington, NY 10533, USA) Measurements were repeated three times; the highest values were recorded and expressed as percentages	Quadriceps femoris muscle strength, mean±sd, N: 166.79±68.29 Quadriceps femoris muscle strength, mean±sd, %: 66.21±19.03 Shoulder abductors muscle strength, median (IQR), N: 107.00 (74.80–127.00) Shoulder abductors muscle strength, median (IQR), %: 78.61 (69.93–107.23) Elbow flexors muscle strength, mean±sd, N: 110.59±46.65 Elbow flexors muscle strength, mean±sd, %: 71.68±19.39 HGS, median (IQR), KgF: 11.00 (8.00–20.00) HGS, median (IQR), %: 65.15 (49.20–80.22)
**Kartal [65], 2024, TR**	Clinical and radiological findings, genotyping, nNO level, TEM, ciliary beat pattern, and frequency criteria	30	13.6±3.5 (mean±sd)	57	HGS	Hand dynamometer (Baseline Standard Hydraulic Hand Dynamometer, 90 kg, Baseline, New York, USA) HGS was measured in a sitting position, with the elbow in 90 degrees of flexion, and the forearm and wrist in a neutral position In order to reduce the effects of muscle fatigue, a 1-min break was given between measurements The hand dynamometer was squeezed with maximum force throughout the test and held for 3 s	HGS, median (IQR), kg, severe/moderate PCD: 16.88 (10.62–25.26) HGS, median (IQR), kg, mild PCD: 13.5 (8.82–18.64) HGS, median (IQR), kg, normal PCD: 24.75 (16.88–40.13)
**Mutlu [60], 2025, TR**	Results consistent with PCD diagnosis in genetic or HSVM according to ATS and ERS guidelines	27	14.11±3.24 (mean±sd)	48	Deltoid muscle strength	Using a hand dynamometer (JTECH Power Track Commander, Baltimore, USA) The assessments for the right and left sides were repeated three times Values were recorded in N and the highest value was selected for analysis The percentage of the expected value for age and sex was used to express measurement results	Deltoid muscle, median (IQR), N, right: 140 (110–175) Deltoid muscle, mean±sd, %, right: 87.52±18.01 Deltoid muscle, median (IQR), N, left: 138 (110–180) Deltoid muscle, mean±sd, %, left: 86.47±19.63

ATS: American Thoracic Society; CA: Canada; ERS: European Respiratory Society; HGS: hand grip strength; HSVM: high-speed video microscopy; IQR: interquartile range; KgF: kilogram force; MAC: mid-arm Circumference; MAMC: mid-arm muscle circumference; MRI: magnetic resonance imaging; MUAC: mid upper arm circumference; nNO: nasal nitric oxide; N: Newtons; NA: not available; TEM: transmission electron microscopy; TR: Türkiye; TSF: triceps skinfold thickness; UK: United Kingdom. **^#^**: Current age is reported, unless otherwise specified. Age is reported in years.

### Nutrition-related parameters

Only four identified publications reported on the nutrition-related parameters in people with PCD (table 3) [23, 32, 39, 42]. The parameters reported in the publications vary considerably, as do the assessment methods.

**TABLE 3:** Characteristics and findings of included studies reporting on nutrition-related parameters in patients with primary ciliary dyskinesia (PCD)

First author, study name [ref.], year, country	Basis for PCD diagnosis	Study participants (n)	Age^#^	Sex (% female)	Nutrition-related parameter	Parameter assessment	Findings (overall and per subgroups, when available)
**Mirra [23], 2015, IT**	Based on the demonstration of abnormal motility and ultrastructural defects of cilia	22	10.5 (2–34) (median (range))	32	Serum 25(OH)D levels	Single determination of blood samples obtained after overnight fast, using the chemiluminescent method (Liasion, DiaSorin, Saluggia, IT) Vitamin D levels were categorised as being sufficient when >30 ng·mL^−1^ (>75 nmol·L^−1^), insufficient between 20 and 30 ng·mL^−1^ (50 and 75 nmol·L^−1^) and deficient when <20 ng·mL^−1^ (<50 nmol·L^−1^)	Serum 25(OH)D, median (range), ng·mL^−1^: 25 (4.8–49) Serum 25(OH)D classification, n (%): Sufficient: 6 (28) Insufficient: 12 (54) Deficient: 4 (18)
**Marino [32], 2019, UK**	Confirmed according to the ERS consensus guidelines	43 in total, 19–36 for our parameters of interest	7.0±3.6 (mean±sd)	49	Energy intake, protein intake, blood levels of vitamins and micronutrients	3-day food diary (two weekdays and one weekend day) Nutritional intake data were assessed using CompEat Pro (Visual Informatics Systems Ltd., Oxon, UK) Dietary intake for energy and protein were compared to the UK Dietary Reference Values using the RNI for protein and EARs for energy Insufficient protein was defined as an intake standard <100% of the LRNI (meeting nutrient requirements for 2.5% of population), sufficient intake was between the LRNI 100% and ≤200% of the RNI and excessive intake ≥200% of the RNI EARs were used with children consuming ≤67% classified as low energy intake, between 67% EAR and 110% as sufficient intake and excessive intake ≥110% of the EAR Using standard laboratory techniques, routine clinical variables of interest were measured including selenium, zinc, copper, folate, vitamin B12 and vitamin D Vitamin B6 concentrations were measured by HPLC with fluorescence detection Vitamin D levels were categorised as insufficient (<50 nmol·L^−1^) and deficient (<30 nmol·L^−1^)	Energy ≤67% EAR, n (%): 0 Energy ≥110% EAR, n (%): 14 (63) Protein <100% of the LRNI, %: 6 Protein <200% of the LRNI, %: 22 Protein ≥200% of the LRNI, %: 72 Vitamin D, mean±sd, nmol·L^−1^: 52.5±29.2 Vitamin D, classification, n (%): Insufficient: 19 (54) Deficient: 9 (26) Selenium, mean±sd, μmol·L^−1^: 1.4±2.3 (ref range 0.2–0.9) Zinc, mean±sd, μmol·L^−1^: 14.2±2.1 (ref range 11–24) Copper, mean±sd, μmol·L^−1^: 20.0±4.2 (ref range 10–22) Ferritin, mean±sd, μg·L^−1^: 24.0±13.4 (ref range 20–200) Folate, mean±sd, nmol·mL^−1^: 13.0±6.6 (ref range 5–21) Vitamin B12, mean±sd, pg·mL^−1^: 623.5±271 (ref range 180–1000) Vitamin B6, mean±sd, μg·L^−1^: 75.7±42.0 (ref range 5–50) Iron, mean±sd, μmol·L^−1^: 12.0±5.3 (ref range 5–31) Transferrin, mean±sd, g·L^−1^: 3.0±0.4 (ref range 1.8–3.3) Transferrin iron saturation, mean±sd, %: 19.5±9.4 (ref range <16) Albumin, mean±sd, g·L^−1^: 41.4±3.5 (ref range 35–45) Calcium, mean±sd, mmol·L^−1^: 2.3±0.1 (ref range 2.20–2.60) Phosphate, mean±sd, mmol·L^−1^: 1.5±0.3 (ref range 1.25–2.10) Magnesium, mean±sd, mmol·L^−1^: 0.9±0.8 (ref range 0.70–1.00)
**King [39], 2021, UK**	NA	25	23.0 (19.0–27.0) (median (IQR))	68	Energy, protein, carbohydrates, fat, iron, calcium, vitamin D	3×24-h dietary recalls (using a multiple pass technique) by a registered dietitian Dietary recall interviews were undertaken face to face at the clinic appointment and then by telephone interview Each dietary recall was coded, and energy, protein, carbohydrate, fat, vitamin D, iron and calcium intakes were calculated A mean of all seven nutrients for each individual patient was then recorded Food records were analysed by the same dietitian using MyFood 24© and intakes compared to the EAR (energy) and RNI (protein, calcium, vitamin D) Macronutrient values were also presented as a proportion of total energy intake	Energy, median (IQR), kcal: 1615 (1161–2352) Energy % EAR, median (IQR), %: 79.0 (66.0–95.5) Protein, median (IQR), g: 70.0 (52.0–84.0) Protein % total energy, median (IQR), %: 15.0 (13.0–19.0) Protein % RNI, median (IQR), %: 116.0 (88.2–170.4) Protein % 1 g·kg^−1^, median (IQR), %: 90.6 (67.8–128.1) Carbohydrate, median (IQR), g: 183.3 (153.3–227.3) Carbohydrate % total energy, median (IQR), %: 44.5 (37.0–49.1) Fat, median (IQR), g: 61.3 (44.2–82.8) Fat % total energy, median (IQR), %: 34 (30.2–38) Iron, median (IQR), mg: 8.0 (6.2–9.5) Iron % RNI, median (IQR), %: 80.4 (57.4–113.2) Calcium, median (IQR), mg: 702.0 (468.5–916.5) Calcium % RNI, median (IQR), %: 100.2 (66.9–124.0) Vitamin D, median (IQR), µg: 1.0 (0.0–3.0) Vitamin D % RNI, median (IQR), %: 10.0 (0.0–30.0)
**Lam,** **CH-PCD [42], 2022, CH**	Confirmed or clinical (*i.e.* strong clinical suspicion and history of neonatal respiratory symptoms, but have not completed the diagnostic algorithm)	74	23 (15–51) (median (IQR))	51	Physician recommendation for caloric intake increase; use of hypercaloric drinks	Questionnaire	Total study population Physician recommendation to increase caloric intake by, n (%): Energy-dense foods: 1 (1) Larger meal portions: 3 (4) Increased meal frequency: 2 (3) Use of hypercaloric drinks in the past 12 months, n (%), yes: 5 (7) Children Physician recommendation to increase caloric intake by, n (%): Energy-dense foods: 1 (1) Larger meal portions: 1 (4) Increased meal frequency: 1 (4) Use of hypercaloric drinks in the past 12 months, n (%), yes: 0 Adults Physician recommendation to increase caloric intake by, n (%): Energy-dense foods: 0 Larger meal portions: 2 (4) Increased meal frequency: 1 (2) Use of hypercaloric drinks in the past 12 months, n (%), yes: 5 (10)

CH: Switzerland; CH-PCD Swiss PCD registry; EAR: estimated average requirement; ERS: European Respiratory Society; HPLC: high-performance liquid chromatography; IQR: interquartile range; IT: Italy; LRNI: lower reference nutrient intake; NA: not available; ref: reference; RNI: reference nutrient intake; UK: United Kingdom. **^#^**: Current age is reported, unless otherwise specified. Age is reported in years.

The most frequently reported parameter was vitamin D insufficiency or deficiency, which was reported in two studies [23, 32] conducted in paediatric (n=1) or mixed populations (n=1). Both studies reported on the high prevalence of vitamin D insufficiency or deficiency in patients with PCD, based on blood measurements. In one study, the period of assessment was from September to April [32], while in the other it was from March to June [23]. The prevalence of insufficiency was 54% in both studies, while deficiency was reported for 18–26% of the study participants. Of note, the studies used different cut-offs to define insufficiency or deficiency, which makes comparison between them challenging.

Three studies assessed the intake of macronutrients and micronutrients (n=2), as well as the prescription of high caloric beverages and physician's advice to increase the caloric intake (n=1). The dietary assessment methods in the studies were a 3-day food diary and three 24-h dietary recalls, respectively, while the latter study used a questionnaire to assess the nutrition-related parameters. The studies reported average to high intake of energy and protein, while for most micronutrients and vitamins, the reported intakes or blood levels were within the normal ranges, except for vitamin D intake, which was well below the recommendation [32, 39]. Few patients with PCD reported that a physician recommended they increase their caloric intake or that they used hypercaloric drinks, mainly adults [42].

## Discussion

In the first systematic review of growth- and nutrition-related parameters in children and adults with PCD, we collated the available scientific evidence and saw variability in growth parameters, a high prevalence of undernutrition in children, a considerable prevalence of obesity and a high prevalence of vitamin D insufficiency or deficiency.

A possible explanation for the variability in growth parameters in patients with PCD could be due to differences in the age at diagnosis. Patients with unfavourable growth parameters could have been diagnosed later in life and not have received any nutritional care until a definite diagnosis of PCD was made and they were referred to a specialist clinic. In many centres, clinical evaluation and advice by a dietician is not available routinely even after diagnosis and deviations in growth parameters might go unnoticed until prominent. Given the association of growth outcomes with better respiratory outcomes and the link between nutrition and immune function, it is imperative to monitor nutrition of patients with suspicion of PCD and provide care and treatment to all patients presenting with impaired growth. Waiting for a definite diagnosis to address impaired growth could lead to lifelong consequences and hinder respiratory outcomes. Furthermore, growth should be monitored and reported throughout all stages of disease progression, including in patients with severe respiratory failure and those undergoing lung transplantation, as evidence is limited [66]. Given that weight and height are routinely measured in patients with PCD as part of the lung function assessment, we urge future studies to include information related to their assessment and the estimation of growth-related z-scores, to allow for comparisons between studies.

We noted a substantial prevalence of undernutrition, defined mostly as BMI z-score≤−2, in children with PCD, which ranged from 4 to 11%; all identified studies were conducted in Europe. Europe-wide undernutrition estimates based on BMI z-scores are not available; however, the percentage of children under 5 years of age with low height for age is estimated to be around 3.6%, while regional differences persist (4.6% in Eastern Europe *versus* 2.5% in Western Europe) [67]. Although such comparison should be interpreted with caution given that the measures compared are not the same, the results we present are alarming, as the prevalence of undernutrition in children with PCD was higher than the European average. Furthermore, underweight ranged from 0 to 50% in the included studies, which often combined data from children and adults. The estimated global age-standardised prevalence of underweight in adults in 2022 was 7% in women and 6.2% in men [68]. For many years, academic studies and policy actions addressing malnutrition primarily targeted obesity, often resulting in undernutrition being overlooked as an outdated issue. Persistent efforts to raise awareness on undernutrition, such as the nutritionDay held annually in hospitals and nursing homes [69], as well as the recent decision to include a separate code for undernutrition in adults (5B72) starting in 2027 in the International Classification of Diseases 11th Revision (ICD-11) [70] will hopefully lead to an increase in clinical assessments, research and policy to address all forms of malnutrition in Europe.

On the other hand, overweight ranged from 3 to 14% and obesity from 4 to 25%, in the included studies, which often combined data from children and adults. In contrast, in 2022, the estimated global age-standardised prevalence of obesity was 18.5% in women and 14% in men [68]. While the prevalence estimates in patients with PCD vary and are in some studies slightly below the global age-standardised estimates, many patients with PCD increase their caloric intake without proper assessment or specific recommendations of healthier nutrient-dense sources. However, obesity alters lung morphology and has a negative impact on respiratory function; it is therefore important to ensure patients with chronic respiratory diseases, including PCD, receive timely nutritional advice to ensure healthy weight.

The consistently reported high prevalence of vitamin D insufficiency or deficiency is concerning and should be addressed. In the included studies, that assessed vitamin D levels from autumn to early summer deficiency was seen in 18–26% of participants. In contrast, country-specific estimates report 6.4–59% deficiency [71–73], but the definitions and age groups differed from those included in the review. Our findings highlight that assessment of vitamin D status should be introduced as part of standard care for PCD, which is also supported by the recent international consensus statement on routine blood testing in PCD [74] and, when needed, supplementation should be introduced as part of the treatment plan for patients with PCD.

Strengths of our study are the extensive systematic search, which also considered not-yet-peer-reviewed literature, increasing the possibility of identifying all relevant articles. Additionally, we screened and assessed all articles identified by two researchers, including the risk of bias assessment. Of note, since our literature search relied on bibliographic databases rather than full-text searching, studies addressing the nutrition or growth of patients with PCD that did not mention relevant terms in the title, abstract or author keyword fields and were not indexed with appropriate subject headings have been missed. It is common for clinical studies to refer to basic growth parameters when describing the overall characteristics of included participants; however, these studies rarely included any further valuable information on the topic that could enrich this review. However, given the considerable number of studies we identified on growth, spanning age groups and geographical locations, we are confident that any unidentified studies would not alter our conclusions. The main limitation of this work is that due to the lack of important information on the assessment of growth-related parameters, the use of different z-score reference standards and the differences in classification in the vitamin D insufficiency or deficiency assessments, no meta-analysis could be conducted.

## Conclusion

This is the first study to collate the available evidence on growth and nutrition in patients with PCD. Given the limited data on the nutritional intake of patients with PCD, studies exploring whole diets, key habits and nutrition-related behaviours, ideally in various populations and multicentred settings are needed. While we found considerable variability in growth-related findings, the findings on undernutrition in children, on the prevalence of obesity and vitamin D insufficiency or deficiency highlight the need for timely and frequent assessment of nutrition and growth, including assessment of vitamin D status as standards of care for PCD. Formal malnutrition screening and vitamin D supplementation should be considered as part of the treatment plan for patients with PCD when deemed necessary.

**Points for clinical practice**

- The findings of this systematic review of growth and nutrition in PCD highlight the need for timely and frequent assessment of nutrition and growth as standards of care in PCD.
- Vitamin D status assessment should be introduced as part of standard care for PCD.
- It is imperative to monitor nutrition of patients with suspicion of PCD and provide care and treatment to all patients presenting with impaired growth.
- Given the limited data on the nutritional intake of patients with PCD, studies exploring whole diets, key habits and nutrition-related behaviours are needed.

## Supplementary material

**Please note:** supplementary material is not edited by the Editorial Office, and is uploaded as it has been supplied by the author. Supplementary methods ERR-0024-2026.SUPPLEMENT Supplementary tables ERR-0024-2026.SUPPLEMENT2 PRISMA 2020 checklist ERR-0024-2026.SUPPLEMENT3
