# Supplementary material for: Nutrition and growth of primary ciliary dyskinesia patients: a systematic review
Source: Eur Respir Rev. 2026 May 27;35(180):260024. doi: 10.1183/16000617.0024-2026 (PMC13213463; doi:10.1183/16000617.0024-2026)
Supplement: Supplementary file 1 [file ERR-0024-2026.SUPPLEMENT.pdf]

Supplementary Methods

Nutrition and growth of primary ciliary dyskinesia (PCD) patients: a systematic review

Nena Karavasiloglou, Vasiliki Gkatzou, Andrea Fernandez Rodriguez, Valérie Schwartz, Myrofora Goutaki

1. Full Search Strategy per Database

What is the nutritional status, nutritional intake and growth in patients with primary ciliary dyskinesia (PCD)?

a) **Overview databases & results**

Date of search: **June 12, 2025**

Database	*Platform*	*Coverage*	*Before deduplication*	*After deduplication*
Medline ALL	Ovid	1946-Present	183	
Embase.com	Elsevier	1974-Present	589	
PubMed	NLM	1946-Present	193	
Scopus	Elsevier	1970-Present	293	
Total			*1258*	*720*

538 duplicate records removed using [1]Deduklick

[1]. Borissov, N.; Haas, Q.; Minder, B.; Kopp-Heim, D.; von Gernler, M.; Janka, H.; Teodoro, D.; Amini, P. Reducing systematic review burden using Deduklick: A novel, automated, reliable, and explainable deduplication algorithm to foster medical research. Syst. Rev. 2022, 11, 172.

b) **Search concepts** (block-building approach)

Medline ALL (via Ovid)
(#1 AND #2 NOT #3 (Filters)

**1 Primary Ciliary Dyskinesia (PCD)**
exp Ciliary Motility Disorders/ or ((primary adj2 ciliary adj2 dyskinesia*) or ((Kartagener* or Siewert*) adj2 syndrom*) or (immotil* adj2 cilia* adj2 syndrom*)).ti,ab,kf.
**2) Nutritional Status**
Nutritional Status/ or Body Mass Index/ or exp Body Weight/ or exp Body Composition/ or exp Body Height/ or exp Diet/ or Eating/ or (nutrition* or micronutrient* or micro-nutrient* or macronutrient* or macro-nutrient* or vitamin* or energy or body mass index or BMI or growth or length or height or stature*).ti,ab,kf
**3) Date limit: 2005-current**
limit x to yr="2005 - 2025"

c) **Reporting of all search strategies**

Medline ALL (via Ovid)
Coverage 1946-Present. We restricted the search to studies published from 2005 to the present.

(exp Ciliary Motility Disorders/ or ((primary adj2 ciliary adj2 dyskinesia*) or ((Kartagener* or Siewert*) adj2 syndrom*) or (immotil* adj2 cilia* adj2 syndrom*)).ti,ab,kf.) AND (Nutritional Status/ or Body Mass Index/ or exp Body Weight/ or exp Body Composition/ or exp Body Height/ or exp Diet/ or Eating/ or (nutrition* or micronutrient* or micro-nutrient* or macronutrient* or macro-nutrient* or vitamin* or energy or body mass index or BMI or growth or length or height or stature*).ti,ab,kf.) AND 2005:2025.(sa_year).

.kf = Keyword Heading Word assigned by authors (author keywords)

Embase.com (via Elsevier)
Coverage 1974-Present. We restricted the search to studies published from 2005 to the present.

('ciliary dyskinesia'/exp OR ((primary NEAR/2 ciliary NEAR/2 dyskinesia*) or ((Kartagener* or Siewert*) NEAR/2 syndrom*) or (immotil* NEAR/2 cilia* NEAR/2 syndrom*)):ti,ab,kw) AND ('nutritional status'/exp OR 'body mass'/de OR 'body weight'/exp OR 'body composition'/exp OR 'body height'/de OR 'diet'/exp OR 'eating'/de OR (nutrition* or micronutrient* or micro-nutrient* or macronutrient* or macro-nutrient* or vitamin* or energy or "body mass index" or BMI or growth or length or height or stature*):ti,ab,kw) AND [2005-2025]/py

PubMed (via NLM)
Coverage 1946-Present. We restricted the search to studies published from 2005 to the present.

("Ciliary Motility Disorders"[Mesh] OR (primary[tiab] AND ciliary[tiab] AND dyskinesia*[tiab]) OR ((Kartagener*[tiab] OR Siewert*[tiab]) AND syndrom*[tiab]) OR (immotil*[tiab] AND cilia*[tiab] AND syndrom*[tiab])) AND ("Nutritional Status"[Mesh] OR "Body Mass Index"[Mesh] OR "Body Weight"[Mesh] OR "Body Composition"[Mesh] OR "Body Height"[Mesh] OR "Diet"[Mesh] OR "Eating"[Mesh] OR nutrition*[tiab] OR micronutrient*[tiab] OR micro-nutrient*[tiab] OR macronutrient*[tiab] OR macro-nutrient*[tiab] OR vitamin*[tiab] OR energy[tiab] OR body mass index[tiab] OR BMI[tiab] OR growth[tiab] OR length[tiab] OR height[tiab] OR stature*[tiab]) AND ("2005/01/01"[dp] : "2025/06/12"[dp])

Scopus (via Elsevier)
Coverage 1970-Present. We restricted the search to studies published from 2005 to the present.

TITLE-ABS-KEY ("ciliary motility disorder*" OR (primary W/2 ciliary W/2 dyskinesia*) OR ((kartagener* OR siewert*) W/2 syndrom*) OR (immotil* W/2 cilia* W/2 syndrom*)) AND TITLE-ABS-KEY (nutrition* OR micronutrient* OR micro-nutrient* OR macronutrient* OR macro-nutrient* OR vitamin* OR energy OR "body mass index" OR bmi OR growth OR length OR height OR stature) AND PUBYEAR > 2004 AND PUBYEAR < 2026

2. Risk of bias assessor guidelines

We developed these guidelines to assist the assessors in their risk of bias evaluation. We based the evaluation on a modified version of the Agency for Healthcare Research and Quality (AHRQ) tool, as cited in Mamikutty, Rokiah et al. 2021 (1). Out of the 11 items of the AHRQ tool mentioned in Mamikutty et al, we retained the 7 items that were relevant to our study question (Items Q1, Q2, Q3, Q4, Q6, Q8, Q9). Questions Q5, Q7, Q10, and Q11 of the tool were excluded (Table 1).

We adapted the questions and the scoring, as necessary, to fit our study question. Of note, we adapted our scoring so that when information was not given, this resulted in a higher scoring (i.e. higher risk of bias), compared to when some information was provided which might have been unclear or insufficient. This is a deviation compared to Mamikutty et al (1), which we felt was appropriate, given that growth-related parameters were rarely the primary outcomes of the identified studies, and studies rarely detailed the assessment method of growth-related parameters and the reference standards they used to estimate z-scores.

The lower the score in each question, the lower the risk of bias. The count of the high-quality answers (i.e., 1s) determines the overall quality of the study (2). We adapted quality was adapted to reflect the number of questions we included. We assessed article quality as follows: low quality = 0-2; moderate quality = 3-5; and high quality = 6-7.

The detailed instructions for assessors were as follows:

Q1: What was the source of the information (Cross-sectional survey, cohort study)?

In this question we evaluate the study design. If the study was prospective, we give it a lower score (i.e., assume it is of higher quality)

Score Q1:

1	Cross- sectional survey or cohort study, that cross-sectionally analyses data on growth or nutrition
2	Retrospective assessment of hospital records, due to the level of uncertainty and the basis in already collected data
3	Study type not mentioned

Q2: Were the inclusion and exclusion criteria clearly defined or were previous publications cited that clearly explain the study procedures (e.g., recruitment)? (Selection bias)

Score Q2:

1	Inclusion and exclusion criteria are clearly mentioned, and study procedures are explained
2	The information provided regarding the inclusion and exclusion criteria OR the study procedures is unclear/ insufficient
3	No information provided regarding the inclusion and exclusion criteria OR the study procedures

Q3: Were subjects consecutively asked to participate? Are the subjects representative of the target population (i.e., people diagnosed with primary ciliary dyskinesia [PCD])? (Selection bias)

Score Q3:

1	Subjects were consecutively asked to participate, and the subjects are representative of the target population (i.e., people diagnosed with PCD)
2	Subjects were not consecutively asked to participate, OR the subjects are NOT representative of the target population (e.g., siblings, during exacerbations, under a specific treatment)
3	The information provided regarding recruitment is unclear/ insufficient

Q4: Was the time period of subject recruitment clearly defined?

Given recent changes in PCD diagnostics, and to be able to make meaningful comparisons, it is important to know the time period during which the study subjects were recruited.

Score Q4:

1	The time period of subject recruitment is clearly defined.
2	The time period of subject recruitment is not clearly defined/not provided.

Q6: How reliable is the basis of the PCD diagnosis?

Score Q6:

1	There is evidence that people with PCD were diagnosed following the latest PCD diagnostic guidelines (e.g., ERS guidelines/ATS guidelines). This includes citing the relevant diagnostic guidelines in the Methods section.
2	There is evidence that people with PCD were diagnosed mainly based on clinical phenotype, without supporting objective test results
3	The information provided regarding the PCD diagnosis is unclear/ insufficient, OR the basis of the diagnosis is not mentioned

Q8: How were out parameters of interest assessed? (Detection bias)

Weight, height/length, BMI: Are the assessments of BMI (or height and weight) and classification/z-score comparator of BMI (or height and weight) clearly stated and standard (e.g. WHO, CDC, country-specific)?

Other growth parameters: Is the assessment of other growth parameters (e.g., body composition) and classification clearly stated and standard?

Nutrition: Is the dietary assessment clearly described? Is the categorization clearly described? Is the blood collection clearly described and according to standards?

Score Q8:

	Weight, height/length, BMI	Other growth parameters	Nutrition
1	Assessment clearly explained (measured on the spot) and the classification/z-score comparator are clearly mentioned/defined. This includes mention in the Methods sections OR the relevant table/figure where the results are presented.	Assessment clearly explained (e.g., MRI, BIA, skinfolds). Any classifications are clearly defined. This includes mention in the Methods sections OR the relevant table/figure where the results are presented.	Assessment clearly explained (e.g., 24-hour dietary recalls, food diaries). Classification of nutrients clearly defined (e.g., % EAR, % RNI). This includes mention in the Methods sections OR the relevant table/figure where the results are presented.<br><br>In the case of blood collection, it is clearly described and conducted according to standards (e.g. fasting state). Any classifications (e.g., deficiency, insufficiency) are clearly defined.
2	Either the assessment method (e.g., only mentions record/ medical charts) OR the classification/ z-score comparator is missing.	Either the assessment method OR the classification definition is missing.	Either the assessment method OR the classification definition is missing.
3	BOTH the assessment method (e.g., only mentions record/ medical charts) AND the classification/ z-score comparator are missing.	BOTH the assessment method AND the classification definition are missing.	BOTH the assessment method AND the classification definition are missing.

Q9: If any, explain any subject exclusions from analysis (Attrition bias)

Were all study participants included in the analysis of our parameters of interest? If not, was appropriate justification provided?

Score Q9:

1	There is evidence that there was no exclusion of participants during the study and data were complete, OR the exclusion of participants (i.e., incomplete data in our parameters of interest) was clearly addressed and reasons were documented
2	There is evidence that there was incomplete data (i.e., total number of study participants differs from analysed number in our parameter(s) of interest) AND information provided regarding any exclusions of participants in our parameters of interest is unclear/ insufficient
3	There is evidence that there was incomplete data (i.e., total number of study participants differs from analysed number in our parameter of interest) AND no information was provided regarding any exclusions of participants in our parameters of interest/unavailability of data.

Questions that were not considered in the study quality score [as described in (1)] and reasons for exclusion

Question	Reason for exclusion
Q5: Indicate if evaluators of subjective components of study were masked to other aspects of the status of the participants. Are the evaluators professional (trained /calibrated)?	The vast majority of the studies included in our systematic review were embedded in clinical care. As such, masking is not relevant, and it is assumed that all evaluators were trained healthcare professionals.
Q7: Describe any assessments undertaken for quality assurance purposes (e.g., test/retest of primary outcome measurements)	In the vast majority the studies included in our systematic review, the parameters measure were not primary or secondary outcomes of the studies.
Q10: Describe how confounding was assessed and/or controlled.	Given that our research question is focused on parameters that were assessed/measured, and no outcomes were estimated using statistical tests, this question is not relevant.
Q11: Summarize patient response rates and completeness of data collection	We decided to include these aspects in our assessment of Q9.

References

1. Mamikutty R, Aly AS, Marhazlinda J. Selecting Risk of Bias Tools for Observational Studies for a Systematic Review of Anthropometric Measurements and Dental Caries among Children. Int J Environ Res Public Health. 2021 Aug 15;18(16):8623.

2. Chen D, Zhi Q, Zhou Y, Tao Y, Wu L, Lin H. Association between Dental Caries and BMI in Children: A Systematic Review and Meta-Analysis. Caries Res. 2018;52(3):230–45.
