# Supplementary material for: Nutrition and growth of primary ciliary dyskinesia patients: a systematic review
Source: Eur Respir Rev. 2026 May 27;35(180):260024. doi: 10.1183/16000617.0024-2026 (PMC13213463; doi:10.1183/16000617.0024-2026)
Supplement: Supplementary file 2 [file ERR-0024-2026.SUPPLEMENT2.pdf]

Supplementary Tables

Nutrition and growth of primary ciliary dyskinesia (PCD) patients: a systematic review

Nena Karavasiloglou, Vasiliki Gkatzou, Andrea Fernandez-Rodriguez, Valérie Schwartz, Myrofora Goutaki

Supplementary Table S1. Risk of bias assessment score of the included studies

First author	Year	Risk of bias assessment score*
Wells [1]	2011	2
Valerio [2]	2012	6
Fowler [3]	2013	4
Svobodová [4]	2013	2
Cohen-Cymberknoh [5]	2014	3
Maglione [6]	2014	3
Mirra [7]	2015	6
Yiallouros [8]	2015	3
Cohen-Cymberknoh [9]	2017	3
Frija-Masson [10]	2017	3
Goutaki [11]	2017	3
Irving [12]	2018	4
Marino [13]	2019	4
Emiralioğlu [14]	2020	5
Piatti [15]	2020	3
Pifferi [16]	2020	4
Rubbo [17]	2020	5
Alzaid [18]	2021	5
Constant [19]	2021	3
Kilinc [20]	2021	2
King [21]	2021	5
Pifferi [22]	2021	5
Verkleij [23]	2021	4
Firat [24]	2022	5
Guan [25]	2022	4
Halbeisen [26]	2022	4
Kos [27]	2022	3
Lam [28]	2022	5
Asseri [29]	2023	4
Cohen-Cymberknoh [30]	2023	3
Fein [31]	2023	4
Gatt [32]	2023	3
Graziano [33]	2023	3
Kinghorn [34]	2023	5
Rumman [35]	2023	5
Zhou [36]	2023	1
Ademhan Tural [37]	2024	4
Ewen [38]	2024	6
Kaspy [39]	2024	5
Kinghorn [40]	2024	6

Koucký [41]	2024	4
Lam [42]	2024	5
Marzook [43]	2024	5
Raidt [44]	2024	4
Rubbo [45]	2024	5
Holgersen [46]	2025	3
Kahraman [47]	2025	4
Macatangay [48]	2025	3
Mutlu [49]	2025	5
Sunman [50]	2025	5

*The article quality score ranged from 0-7, with higher score reflecting higher quality. Furthermore, we classified the score as follows: low quality = 0-2; moderate quality = 3-5; and high quality = 6-7

Supplementary Table S2. Characteristics and findings of included studies reporting on the height or length, weight, and BMI in patients with PCD

First author, Study name	Year	Country	Basis for PCD diagnosis	Study participants (n)	Age *	Sex (% female)	Parameter assessment	Growth comparator	Findings (overall and per subgroups, when available)
Wells [1]	2011	CA	NA	10	13.8 ± 2.3, mean ± SD	40	Height and weight measured (model 555; SR Instruments, Tonawanda, NY).	NA	Height, mean ± SD, m: 1.6 ± 0.1<br>Mass, mean ± SD, kg: 51.2 ± 11.2
Valerio [2]	2012	IT	Light microscopy and electron microscopic ultrastructural analysis of cilia	10	13.2 ± 2.8, mean ± SD	30	Height and weight were measured with the subjects wearing only light clothes and no shoes	IT data	Total study population<br>BMI, mean ± SD, kg/m^2: 21.1 ± 3.05<br>BMI-SDS, mean ± SD: 0.24 ± 0.91<br>Preserved lung function<br>BMI, mean ± SD, kg/m^2: 22.6 ± 3.1<br>BMI-SDS, mean ± SD: 0.60 ± 1.0<br>Reduced lung function<br>BMI, mean ± SD, kg/m^2: 18.9 ± 0.8<br>BMI-SDS, mean ± SD: - 0.31 ± 0.42
Fowler [3]	2013	US	PCD was determined by electron microscopy or definitive clinical manifestations associated with low nNO production and evidence of ciliary dysfunction	8	36 ± 17.6, mean ± SD	88	NA	NA	BMI, mean ± SD: 22.3 ± 8.3
Svobodová [4]	2013	CZ	The diagnostic criteria were: (1) clinical findings consistent with a diagnosis of PCD (otosinopulmonary symptoms); (2) abnormal	29 in total, 12-25, depending on the timing of assessment	14.5 [1.2-24], median [range]	55, total study population	Body height was measured using a wall-mounted stadiometer according to routine medical practice, and body length in children aged 1 year	CZ data	Total study population<br>Length/height, mean SDS ± SEM, 1 year: 0.40 ± 0.24<br>Length/height, mean SDS ± SEM, 3 years: 0.16 ± 0.23<br>Length/height, mean SDS ± SEM, 5 years: -0.13 ± 0.21

First author, Study name	Year	Country	Basis for PCD diagnosis	Study participants (n)	Age *	Sex (% female)	Parameter assessment	Growth comparator	Findings (overall and per subgroups, when available)
Svobodová [4] (cont.)			video/HSVM of cilia confirmed at least 2 times with an interval between examinations of at least 4-6 weeks; (3) TEM findings consistent with a ciliary movement abnormality.				old was measured using a bodymeter		Length/height, mean SDS ± SEM, 7 years: -0.54 ± 0.19<br>Length/height, mean SDS ± SEM, 9 years: -0.67 ± 0.21<br>Length/height, mean SDS ± SEM, 11 years: -0.52 ± 0.24<br>Length/height, mean SDS ± SEM, 13 years: -0.53 ± 0.23<br>Length/height, mean SDS ± SEM, 15 years: -0.53 ± 0.29<br>Length/height, mean SDS ± SEM, 17 years: -0.67 ± 0.38<br>Study participants with known mutations<br>Length/height, mean SDS ± SEM, 1 year: 0.17 ± 0.36<br>Length/height, mean SDS ± SEM, 3 years: 0.00 ± 0.41<br>Length/height, mean SDS ± SEM, 5 years: -0.54 ± 0.38<br>Length/height, mean SDS ± SEM, 7 years: -0.72 ± 0.31<br>Length/height, mean SDS ± SEM, 9 years: -0.91 ± 0.30<br>Length/height, mean SDS ± SEM, 11 years: -0.92 ± 0.46<br>Length/height, mean SDS ± SEM, 13 years: -0.78 ± 0.46<br>Length/height, mean SDS ± SEM, 15 years: -0.68 ± 0.60<br>Length/height, mean SDS ± SEM, 17 years: -0.96 ± 0.56

First author, Study name	Year	Country	Basis for PCD diagnosis	Study participants (n)	Age *	Sex (% female)	Parameter assessment	Growth comparator	Findings (overall and per subgroups, when available)
Svobodová [4] (cont.)									Total study population<br>BMI, mean SDS ± SEM, 1 year: -0.29 ± 0.25<br>BMI, mean SDS ± SEM, 3 years: -0.42 ± 0.33<br>BMI, mean SDS ± SEM, 5 years: -0.02 ± 0.24<br>BMI, mean SDS ± SEM, 7 years: -0.26 ± 0.20<br>BMI, mean SDS ± SEM, 9 years: -0.30 ± 0.18<br>BMI, mean SDS ± SEM, 11 years: -0.10 ± 0.21<br>BMI, mean SDS ± SEM, 13 years: -0.25 ± 0.16<br>BMI, mean SDS ± SEM, 15 years: -0.21 ± 0.29<br>BMI, mean SDS ± SEM, 17 years: -0.38 ± 0.23<br>Study participants with known mutations<br>BMI, mean SDS ± SEM, 1 year: -0.71 ± 0.54<br>BMI, mean SDS ± SEM, 3 years: -0.51 ± 0.93<br>BMI, mean SDS ± SEM, 5 years: -0.11 ± 0.60<br>BMI, mean SDS ± SEM, 7 years: -0.32 ± 0.33<br>BMI, mean SDS ± SEM, 9 years: -0.37 ± 0.31<br>BMI, mean SDS ± SEM, 11 years: -0.10 ± 0.26<br>BMI, mean SDS ± SEM, 13 years: -0.29 ± 0.22

First author, Study name	Year	Country	Basis for PCD diagnosis	Study participants (n)	Age *	Sex (% female)	Parameter assessment	Growth comparator	Findings (overall and per subgroups, when available)
Svobodová [4] (cont.)									BMI, mean SDS ± SEM, 15 years: -0.50 ± 0.32<br>BMI, mean SDS ± SEM, 17 years: -0.51 ± 0.34
Cohen-Cymberknoh [5]	2014	IS	Typical clinical presentation together with low nNO and abnormal ciliary morphology on electron microscopy and/or abnormal ciliary beat pattern and/or two PCD known mutations	34	15.9 ± 8.6, mean ± SD	59	NA	European data	BMI % ± SD: 21.3 ± 19.3
Maglione [6]	2014	International	Confirmed diagnosis according to 2009 ERS consensus statement	78-158, depending on the length of follow-up	Age at first spirometry: 8.7 [4.2-17.4], median [range]	49	Measured, NOS	UK data	Height, median [range], cm, 1st measurement: 128.9 [99.5-184] Height, mean ± SD, z-score, 1st measurement: -0.05 ± 1.11<br>Height, median [range], cm, Year 2: 137.3 [111-186]<br>Height, mean ± SD, z-score, Year 2: -0.06 ± 1.11<br>Height, median [range], cm, Year 4: 148.8 [123-194]<br>Height, mean ± SD, z-score, Year 4: 0.10 ± 1.11<br>Height, median [range], cm, Year 6: 159.8 [132-195],<br>Height, mean ± SD, z-score, Year 6: 0.13 ± 1.01<br>Weight, median [range], kg, 1st measurement: 27 [13.5-100]

First author, Study name	Year	Country	Basis for PCD diagnosis	Study participants (n)	Age *	Sex (% female)	Parameter assessment	Growth comparator	Findings (overall and per subgroups, when available)
Maglione [6] (cont.)									Weight, mean ± SD, z-score, 1st measurement: 0.01 ± 1.19<br>Weight, median [range], kg, Year 2: 33.7 [18.7-103]<br>Weight, mean ± SD, z-score, Year 2: 0.06 ± 1.23<br>Weight, median [range], kg, Year 4: 41.5 [23-103]<br>Weight, mean ± SD, z-score, Year 4: 0.24 ± 1.14<br>Weight, median [range], kg, Year 6: 50.5 [29.9-114]<br>Weight, mean ± SD, z-score, Year 6: 0.32 ± 1.08<br>BMI, median [range], kg/m^2, 1st measurement: 16.5 [11.5-32.6]<br>BMI, mean ± SD, z-score, 1st measurement: 0.03 ± 1.27<br>BMI, median [range], kg/m^2, Year 2: 17.3 [11.6-33.3]<br>BMI, mean ± SD, z-score, Year 2: 0.12 ± 1.31<br>BMI, median [range], kg/m^2, Year 4: 18.5 [13.4-33.6]<br>BMI, mean ± SD, z-score, Year 4: 0.25 ± 1.22<br>BMI, median [range], kg/m^2, Year 6:19.5 [14.1-37.2]<br>BMI, mean ± SD, z-score, Year 6: 0.29 ± 1.23<br>BMI <1.96, n (%), 1st measurement: 11 (7%)<br>BMI >1.96, n (%), 1st measurement: 10 (6%)

First author, Study name	Year	Country	Basis for PCD diagnosis	Study participants (n)	Age *	Sex (% female)	Parameter assessment	Growth comparator	Findings (overall and per subgroups, when available)
Mirra [7]	2015	IT	Based on the demonstration of abnormal motility and ultrastructural defects of cilia	22	10.5 [2-34], median [range]	32	NA	NA	BMI, median [range], kg/m^2: 18.5 [13-37]<br>Vitamin D deficiency-to-insufficiency and sufficiency groups did not show any significant differences in BMI (19 versus 17 kg/m^2, p = 0.7)
Yiallouros [8]	2015	CY	Abnormal TEM findings. If TEM findings were normal, then both abnormal HSVM and low nNO levels were required	30	24.3 [0.7-63.7], median [range]	50	NA	CDC data	<u>Total study population</u><br>BMI, median [range], z-score, at presentation: -0.57 [-2.25-1.35]<br><u>Study participants with presentation <18 years</u><br>BMI, median [range], z-score, at presentation: 0.03 [-1.21-0.93]<br><u>Study participants with presentation >18 years</u><br>BMI, median [range], z-score, at presentation, overall: -0.7 [-2.25-1.35]<br>BMI, median [range], z-score, at presentation, no lobectomy: 0.13 [-2.16-1.35]<br>BMI, median [range], z-score, at presentation, with lobectomy: -1.57 [-2.25 - -0.7]
Cohen-Cymberknoh [9]	2017	International	ERS guidelines	217 in total, 159 for our parameters of interest	Age at the end of the study period: 19.9 ± 13.9 [0-67], median ± SD [range]	NA	Retrieved from patient records	NA	<u>Total study population</u><br>BMI, mean ± SD [range], percentile, 0-20 years: 44.0 ± 29.9 [1-98]<br>BMI, mean ± SD [range], kg/m^2, >20 years: 22.6 ± 3.85 [16.9-37]<br><u>Colonised</u><br>BMI, mean ± SD [range], percentile, 0-20 years: 41 ± 30 [3-97]<br>BMI, mean ± SD [range], kg/m^2, >20 years: 22.7 ± 3.8 [16.9-37]

First author, Study name	Year	Country	Basis for PCD diagnosis	Study participants (n)	Age *	Sex (% female)	Parameter assessment	Growth comparator	Findings (overall and per subgroups, when available)
Cohen-Cymberknoh [9] (cont.)									Non-colonised<br>BMI, mean ± SD [range], percentile, 0-20 years: 44.8 ± 29 [1-98]<br>BMI, mean ± SD [range], kg/m^2, >20 years: 22.5 ± 3.4 [17.2-33.3]
Frija-Masson [10]	2017	FR	At least one of the following: (1) a clinical diagnosis of Kartagener syndrome (i.e., chronic sinusitis, bronchiectasis and situs inversus) and (2) the evidence of PCD-specific ciliary ultrastructural defects or the identification of non-ambiguous causal mutations in a PCD gene in a patient with bronchiectasis on CT scan	78	[18-77], [range]	41	NA	NA	Total study population<br>BMI, median (25-75): 22.9 (20.8-26.1)<br>Females<br>BMI, median (25-75): 22.6 (20.1-26.4)<br>Males<br>BMI, median (25-75): 23.4 (21.7-26.1)

First author, Study name	Year	Country	Basis for PCD diagnosis	Study participants (n)	Age *	Sex (% female)	Parameter assessment	Growth comparator	Findings (overall and per subgroups, when available)
Goutaki, iPCD [11]	2017	International	Patients were divided into three diagnostic subgroups. The first subgroup included patients with definite PCD, which was defined, based on recent guidelines of the ERS PCD Diagnostics Task Force, by hallmark TEM findings and/or an identified biallelic PCD genetic mutation. The second subgroup, probable PCD, included patients with abnormal HSVM findings and/or low nNO. The third subgroup included patients with a clinical PCD diagnosis	1549-1609, depending on the parameter	[0 - >50], [range]	51	NA	WHO and national data	Total study population<br>Height, mean (95% CI), z-score, comparison with national data: -0.27 (-0.33, -0.21)<br>Height, mean (95% CI), z-score, comparison with WHO data: -0.12 (-0.17, -0.06)<br>Paediatric (<20 years)<br>Height, mean (95% CI), z-score, comparison with national data: -0.26 (-0.33, -0.20)<br>Adult (⩾20 years)<br>Height, mean (95% CI), z-score, comparison with national data: -0.31 (-0.42, -0.20)<br>Definite PCD<br>Height, mean (95% CI), z-score, comparison with national data: -0.26 (-0.33, -0.19)<br>Total study population<br>BMI, mean (95% CI), z-score, comparison with national data: 0.06 (0.002, 0.13)<br>BMI, mean (95% CI), z-score, comparison with WHO data: 0.21 (0.14, 0.27)<br>Underweight (BMI z-score ≤ -2), n (%): 105 (6)<br>Obesity (BMI z-score ≥ 2), n (%): 405 (25)<br>Paediatric (<20 years)<br>BMI, mean (95% CI), z-score, comparison with national data: 0.02 (-0.05, 0.09)

First author, Study name	Year	Country	Basis for PCD diagnosis	Study participants (n)	Age *	Sex (% female)	Parameter assessment	Growth comparator	Findings (overall and per subgroups, when available)
Goutaki, iPCD [11] (cont.)									Definite PCD<br>BMI, mean (95% CI), z-score, comparison with national data: 0.05 (-0.02, 0.13)
Irving [12]	2018	UK	A positive or highly likely diagnosis of PCD according to current European guidelines based on:<br>1) a hallmark TEM defect.<br>2) biallelic changes in a known PCD gene<br>3) consistent reproducible defects on HSVM, coupled with persistently low nNO and clinical features of PCD	69	13 [4-41], median [range]	64	NA	NA	Total study population<br>BMI, median [range], kg/m^2: 19.4 [10.7-27.5]<br>Participants with normal ultrastructure<br>BMI, median [range], kg/m^2: 18.9 [13.2-24.0]<br>Participants with ODA ± IDA<br>BMI, median [range], kg/m^2: 20.8 [12.9-27.5]<br>Participants with MTD<br>BMI, median [range], kg/m^2: 18.7 [10.7-25.9]
Marino [13]	2019	UK	Confirmed according to the ERS consensus guidelines	43 in total, 26-43 for our parameters of interest	7.0 ± 3.6, mean ± SD	49	Infants aged ≤1 year were weighed naked and children aged ≥1 year with minimal clothing; weight was measured to the nearest 0.1 kg using a digital scale. Recumbent length was measured to the nearest 0.1 cm for all children aged 2	WHO data	Height, mean ± SD, m: 1.2 ± 0.3<br>Height for age, mean ± SD, z-score: -0.2 ± 1.1<br>Weight, mean ± SD, kg: 30.3 ± 19.8<br>Weight for age, mean ± SD, z-score: 0.1 ± 1.1<br>BMI, mean ± SD, kg/m^2: 17.2 ± 4.6<br>Moderate malnutrition (Height for age z-score ≤ -2), n (%): 2 (4.6)<br>Moderate malnutrition (BMI z-score ≤ -2), n (%): 3 (6.9)

First author, Study name	Year	Country	Basis for PCD diagnosis	Study participants (n)	Age *	Sex (% female)	Parameter assessment	Growth comparator	Findings (overall and per subgroups, when available)
Marino [13] (cont.)							years using an infantometer (Seca 416; Birmingham, UK) and standing in older children under a stadiometer (Seca 213: Birmingham, UK)		
Emiralioğlu [14]	2020	TR	Clinical phenotype characteristics, ciliary ultrastructural defects (using TEM), abnormal ciliary function (using HSVM) and genetic pathogenic variants	46	Age at diagnosis: 8.5 (0.5-15), median (min-max)	63	Recorded at the last clinical follow-up	WHO data	<u>Total study population</u><br>BMI, median (min-max), kg/m^2: 18.5 (13.2-26.1)<br>BMI, median (min-max), z-score: -0.29 (-2.34-2.29)<br><u>Participants with DNAH5 pathogenic variants</u><br>BMI, median (min-max), kg/m^2: 19.1 (15-25.5)<br>BMI, median (min-max), z-score: 0.08 (-2.34-1.19)<br><u>Participants with CCDC40 pathogenic variants</u><br>BMI, median (min-max), kg/m^2: 18.7 (13.2-23)<br>BMI, median (min-max), z-score: -0.78 (-2.33-2.29)<br><u>Participants with RSPH4A pathogenic variants</u><br>BMI, median (min-max), kg/m^2: 20.2 (16.4-24.5)<br>BMI, median (min-max), z-score: -0.06 (-0.97-0.88)<br><u>Participants with DNAH11 pathogenic variants</u><br>BMI, median (min-max), kg/m^2: 21.5 (16-22.3)

First author, Study name	Year	Country	Basis for PCD diagnosis	Study participants (n)	Age *	Sex (% female)	Parameter assessment	Growth comparator	Findings (overall and per subgroups, when available)
Emiralioğlu [14] (cont.)									BMI, median (min-max), z-score: 0.54 (-1.21-0.71)<br>Participants with HYDIN pathogenic variants<br>BMI, median (min-max), kg/m^2: 16 (16-24.6)<br>BMI, median (min-max), z-score: -0.2 (-2.12-0.91)<br>Participants with CCNO pathogenic variants<br>BMI, median (min-max), kg/m^2: 16 (13.6-18)<br>BMI, median (min-max), z-score: -1.2 (-2.2-0.4)<br>Participants with DNAI1 pathogenic variants<br>BMI, median (min-max), kg/m^2: 16.7 (14.3-19)<br>BMI, median (min-max), z-score: -1.01 (-1.26-0.7)<br>Participants with ARMC4 pathogenic variants<br>BMI, median, kg/m^2: 26.1<br>BMI, median, z-score: 1.39<br>Participants with TTC25 pathogenic variants<br>BMI, median, kg/m^2: 16.3<br>BMI, median, z-score: -0.51<br>Participants with DNAH1 pathogenic variants<br>BMI, median, kg/m^2: 18.6<br>BMI, median, z-score: -0.8

First author, Study name	Year	Country	Basis for PCD diagnosis	Study participants (n)	Age *	Sex (% female)	Parameter assessment	Growth comparator	Findings (overall and per subgroups, when available)
Emiralioğlu [14] (cont.)									Participants with CCDC39 pathogenic variants<br>BMI, median, kg/m^2: 19.2<br>BMI, median, z-score: -0.2
Piatti [15]	2020	IT	Presence of hallmark ciliary electron microscopy defect, and/or in case of identification of non-ambiguous biallelic mutations in known PCD-associated genes (patients “PCD positive”)"; Patients with a clinical suspicious of PCD who do not fulfil at least one of the above mentioned criteria to make a certain diagnosis (e.g. patients with normal cilia ultrastructure at TEM and/or patients with no evidence of unambiguous biallelic mutations in known PCD associated genes)	58	Children: 11.1 ± 4.6, mean ± SD; adults: 39.4 ± 14.4, mean ± SD	55	NA	NA	BMI, mean ± SD, kg/m^2, ≥ 2 exacerbations/year: 21.6 ± 4.6<br>BMI, mean ± SD, kg/m^2, < 2 exacerbations/year: 18.6 ± 4.2

First author, Study name	Year	Country	Basis for PCD diagnosis	Study participants (n)	Age *	Sex (% female)	Parameter assessment	Growth comparator	Findings (overall and per subgroups, when available)
Piatti [15] (cont.)			were considered as “highly likely PCD” by the result of the combination of other diagnostic test, e.g. repeated low levels of nNO, and abnormal ciliary motility, according to ERS Guidelines						
Pifferi [16]	2020	IT	Confirmed based on the ERS guidelines. Specifically, 35 PCD-causing genes were tested by next generation sequencing and determined the carrier status in the parents to confirm that the mutations in determining PCD were biallelic. Two ciliary evaluations were carried out to confirm IDA defects	135	Age at enrolment, children: 10.24 ± 3.78, mean ± SD;<br>Age at enrolment, adults: 34.33 ± 10.58, mean ± SD	NA	NA	CDC data	Children<br>BMI, mean ± SD, z-score, overall: 0.10 ± 1.35<br>BMI, mean ± SD, z-score, ODA/IDA: 0.21 ± 1.19<br>BMI, mean ± SD, z-score, IDA/CA/MTD: -0.20 ± 0.95<br>BMI, mean ± SD, z-score, CA: -0.76 ± 2.09<br>BMI, mean ± SD, z-score, ODA: 0.48 ± 1.42<br>BMI, mean ± SD, z-score, normal EM: 0.44 ± 0.94<br>Adults<br>BMI, mean ± SD, kg/m^2, overall: 22.97 ± 3.48<br>BMI, mean ± SD, kg/m^2, ODA/IDA: 22.78 ± 3.23<br>BMI, mean ± SD, kg/m^2, IDA/CA/MTD: 22.87 ± 3.97<br>BMI, mean ± SD, kg/m^2, CA: 22.31 ± 2.93<br>BMI, mean ± SD, kg/m^2, ODA: 23.91 ± 3.50

First author, Study name	Year	Country	Basis for PCD diagnosis	Study participants (n)	Age *	Sex (% female)	Parameter assessment	Growth comparator	Findings (overall and per subgroups, when available)
Pifferi [16] (cont.)									BMI, mean ± SD, kg/m^2, normal EM: 22.37 ± 2.82
Rubbo [17]	2020	UK	Confirmed by a specialist PCD diagnostic multi-disciplinary team, in line with ERS consensus guidelines	333 in total, 310-159 for our parameters of interest	9.8 (5.5-13.8), median (IQR)	47	Height and weight in accordance with WHO guidelines. BMI z-scores were calculated using the WHO Anthro Plus software V.3.2.2.	WHO	Height, mean ± SD, z-score: -0.3 ± 1.1<br>Weight, mean ± SD, z-score: -0.1 ± 1.3<br>BMI, mean ± SD, z-score: 0 ± 1.4
Alzaid [18]	2021	SA	Pathogenic/likely pathogenic biallelic variants in one of the PCD-associated genes	18	Age at last follow-up: 9.2, mean	NA	NA	NA	Total study population<br>BMI, median, kg/m^2: 15.87<br>BMI, median, z-score: -1.48<br>Participants with situs solitus<br>BMI, mean: 14.3<br>Participants with situs inversus<br>BMI, mean: 16.7
Constant [19]	2021	PT	NA	6	13.8 [11-19.2], median [range]	67	Measured, NOS	WHO data	BMI, median [range], z-score: -0.3 [-1.5-0.5]
Kilinc [20]	2021	TR	PICADAR ≥6	14	Age at diagnosis: 9 (3-16), median (min-max)	43	Recorded during follow-up, NOS	NA	Total study population<br>BMI, median [range], z-score: -0.22 [-2.90-1.62]<br>Participants with DNAH9 pathogenic variants<br>BMI, median [range], z-score: -1 [-2.56-1.71]<br>Participants with CCDC40 pathogenic variants<br>BMI, median [range], z-score: -2.13 [-2.90-1.62]

First author, Study name	Year	Country	Basis for PCD diagnosis	Study participants (n)	Age *	Sex (% female)	Parameter assessment	Growth comparator	Findings (overall and per subgroups, when available)
Kilinc [20] (cont.)									Participants with DNAH5 pathogenic variants<br>BMI, median, z-score: 0.13<br>Participants with DNAH1 pathogenic variants<br>BMI, median [range], z-score: -1.9 [-0.34-0.72]<br>Participants with DNAH11 pathogenic variants<br>BMI, median [range], z-score: 0.19 [0.41-0.42]<br>Participants with RSPH4A pathogenic variants<br>BMI, median, z-score: 0.415<br>Participants with HYDIN pathogenic variants<br>BMI, median, z-score: -1.54<br>Participants with ARMC4 pathogenic variants<br>BMI, median [range], z-score: 0.80 [-2.90-1.62]
King [21]	2021	UK	NA	25	23.0 (19.0-27.0), median (IQR)	68	Weight and height were collected using calibrated SECA weighing scales and Leicester Height measure	NA	Weight, median (IQR), kg: 60.9 (51.7-68.3)<br>BMI, median (IQR), kg/m^2: 22.1 (20.6-25.4)
Pifferi [22]	2021	IT	Some or all of the following: ultrastructural and/or functional ciliary abnormalities, in vitro ciliogenesis and/or biallelic	122 in total, 118 for our parameters of interest	Age at enrolment: 25.15 ± 13.9, mean ± SD	53	Measured, NOS	CDC data and WHO categories	Total study population<br>BMI, n (%), at enrolment, overall:<br>Underweight (z-scores ≤ -1.96 or BMI ≤ 18.5): 10 (8.5)<br>Normal: 89 (75.4)<br>Overweight (z-scores ≥ 1.96 or BMI ≥ 25.0): 19 (16.1)

First author, Study name	Year	Country	Basis for PCD diagnosis	Study participants (n)	Age *	Sex (% female)	Parameter assessment	Growth comparator	Findings (overall and per subgroups, when available)
Pifferi [22] (cont.)			PCD disease-causing mutations and nNO combined with compatible clinical features						BMI, n (%), at enrolment, ODA/IDA:<br>Underweight: 2 (9.1)<br>Normal: 15 (71.4)<br>Overweight: 4 (18.2)<br>BMI, n (%), at enrolment, IDA/CA/MTD:<br>Underweight: 4 (12.9)<br>Normal: 22 (71)<br>Overweight: 5 (16.1)<br>BMI, n (%), at enrolment, CA:<br>Underweight: 1 (7.7)<br>Normal: 11 (84.6)<br>Overweight: 1 (7.7)<br>BMI, n (%), at enrolment, ODA:<br>Underweight: 1 (3.8)<br>Normal: 19 (70.3)<br>Overweight: 7 (26.9)<br>BMI, n (%), at enrolment, normal EM:<br>Underweight: 1 (3.8)<br>Normal: 22 (84.6)<br>Overweight: 3 (11.5)<br>Children<br>BMI, mean ± SD, z-score, at enrolment, overall: 0.28 ± 1.21<br>BMI, mean ± SD, z-score, at enrolment, ODA/IDA:<br>0.20 ± 1.60<br>BMI, mean ± SD, z-score, at enrolment, IDA/CA/MTD: -0.2 ± 1.07<br>BMI, mean ± SD, z-score, at enrolment, CA: 0.20 ± 1.74<br>BMI, mean ± SD, z-score, at enrolment, ODA: 0.92 ± 0.80<br>BMI, mean ± SD, z-score, at enrolment, normal EM: 0.29 ± 1.00

First author, Study name	Year	Country	Basis for PCD diagnosis	Study participants (n)	Age *	Sex (% female)	Parameter assessment	Growth comparator	Findings (overall and per subgroups, when available)
Pifferi [22] (cont.)									Adults<br>BMI, mean ± SD, kg/m^2, at enrolment, overall: 22.97 ± 3.51<br>BMI, mean ± SD, kg/m^2, at enrolment, ODA/IDA:<br>22.40 ± 3.41<br>BMI, mean ± SD, kg/m^2, at enrolment, IDA/CA/MTD: 22.86 ± 4.08<br>BMI, mean ± SD, kg/m^2, at enrolment, CA: 22.31 ± 3.21<br>BMI, mean ± SD, kg/m^2, at enrolment, ODA: 23.93 ± 3.45<br>BMI, mean ± SD, kg/m^2, at enrolment, normal EM: 22.37 ± 2.97
Verkleij [23]	2021	NL	Based on combination of tests: HSVM before and after cell culture, nNO, TEM and genetics	57	Children: 13.3±3.3 [7-17], mean ± SD [range]; adults: 38.3±18.0 [18-77], mean ± SD [range]	60	Height and weight were obtained from chart review	NA	BMI, mean ± SD, kg/m^2, children: 20.1 ± 4.4<br>BMI, mean ± SD, kg/m^2, adults: 24.2 ± 4.2
Firat [24]	2022	TR	HSVM or TEM or genetic testing in addition to typical clinical symptoms	27	10.74 ± 4.01, mean ± SD	59	Recorded, NOS	WHO	Height, mean ± SD, z-score:<br>0.02 ± 1.30<br>Weight, mean ± SD, z-score:<br>-0.12 ± 1.17<br>BMI, mean ± SD, z-score:<br>- 0.22 ± 1.28<br>Malnutrition (BMI z-score ≤ -2), n (%): 3 (11.1%)

First author, Study name	Year	Country	Basis for PCD diagnosis	Study participants (n)	Age *	Sex (% female)	Parameter assessment	Growth comparator	Findings (overall and per subgroups, when available)
Guan [25]	2022	CN	Ultrastructural defect or biallelic pathogenic variant; or 2/4 clinical features: unexplained neonatal respiratory distress, year-round daily cough or nasal congestion beginning before 6 months, organ laterality defect and nNO level; or Kartagener syndrome	71	Age at diagnosis: 7.6 ± 3.3, mean ± SD	Discrepant information given in the manuscript. Either 45 or 39, depending on the information used	Collected via electronic medical records	NA	AZM-treated<br>Height, mean ± SD, percentile, baseline: 45.9 ± 27.9<br>Changes of height, median (25-75), percentile, follow-up: 9.6 (−0.2-22.0)<br>Changes of height, n (%), percentile, follow-up:<br>Increased: 18 (75)<br>Decreased: 6 (25)<br>Weight, mean ± SD, percentile, baseline: 33.1 ± 31.0<br>Changes of weight, median (25-75), percentile, follow-up: 5.1 (−0.0-22.0)<br>BMI, mean ± SD, percentile, baseline: 20.7 ± 28.6<br>Changes of BMI, median (25-75), percentile, follow-up: 1.5 (1.0-2.0)<br>BMI, mean ± SD, z-score, baseline: -1.67 ± 1.82<br>Changes of BMI, mean ± SD, z-score, follow-up: 0.44 ± 1.0<br>AZM-untreated<br>Height, mean ± SD, percentile, baseline: 55.7 ± 28.6<br>Changes of height, median (25-75), percentile, follow-up: 6.9 (-17.8-24.5)<br>Changes of height, n (%), percentile, follow-up:<br>Increased: 14 (64)<br>Decreased: 8 (36)<br>Weight, mean ± SD, percentile, baseline: 36.8 ± 26.9<br>Changes of weight, median (25-75), percentile, follow-up: 10.2 (-3.0-28.2)

First author, Study name	Year	Country	Basis for PCD diagnosis	Study participants (n)	Age *	Sex (% female)	Parameter assessment	Growth comparator	Findings (overall and per subgroups, when available)
Guan [25] (cont.)									BMI, mean ± SD, percentile, baseline: 24.2 ± 26.3<br>Changes of BMI, median (25-75), percentile, follow-up: 1.7 (-6.4-19.7)<br>BMI, mean ± SD, z-score, baseline: -1.14 ± 1.18<br>Changes of BMI, mean ± SD, z-score, follow-up: 0.67 ± 1.4
Halbeisen, iPCD [26]	2022	International	Distinguished three levels of diagnostic certainty: patients with definite PCD according to the ERS guidelines with a hallmark ultrastructural defect identified by TEM or pathogenic biallelic PCD genetic mutations; patients with probable PCD who had abnormal HSVM findings or low nNO; and patients diagnosed on clinical grounds with an incomplete diagnostic algorithm	486	Age at diagnosis: 8.64 ± 6.1, mean ± SD	49	NA	WHO data	<u>Total study population</u><br>BMI, mean ± SD, z-score, first measure: -0.04 ± 1.27<br>BMI, mean ± SD, z-score, last measure: 0.01 ± 1.21<br><u>By lung function trajectories</u><br>BMI, mean ± SD, z-score, first measure, improving: -0.2 ± 1.25<br>BMI, mean ± SD, z-score, last measure, improving: 0.02 ± 1.2<br>BMI, mean ± SD, z-score, first measure, stable: 0 ± 1.32<br>BMI, mean ± SD, z-score, last measure, stable: 0.13 ± 1.18<br>BMI, mean ± SD, z-score, first measure, decreasing: 0 ± 1.23<br>BMI, mean ± SD, z-score, last measure, decreasing: -0.13 ± 1.24

First author, Study name	Year	Country	Basis for PCD diagnosis	Study participants (n)	Age *	Sex (% female)	Parameter assessment	Growth comparator	Findings (overall and per subgroups, when available)
Kos [27]	2022	NL	HSVM, TEM, genetics, and nNO	33	[1-71], [range]	55	NA	WHO data	BMI, mean ± SD, kg/m^2: 21.1 ± 3.8<br>BMI, mean ± SD, z-score: 0.05 ± 0.38<br>BMI classification, n (%), no classification definition provided:<br>Underweight: 0 (0)<br>Normal: 26 (88)<br>Overweight: 1 (3)<br>Unknown: 3 (9)
Lam, CH-PCD [28]	2022	CH	Confirmed or clinical (i.e., strong clinical suspicion and history of neonatal respiratory symptoms, but have not completed the diagnostic algorithm)	74	23 (15-51), median (IQR)	51	Height and weight data measured in the hospital or private practices. Values used were the closest to the time when participants completed the survey	WHO data	<u>Total study population</u><br>BMI, n (%):<br>Thinness/underweight: 3 (4)<br>Normal weight: 37 (50)<br>Overweight: 10 (14)<br>Obesity: 3 (4)<br>Missing: 21 (28)<br><u>Children</u><br>BMI, median (IQR), z-score: 1.0 (1.0-1.0)<br>BMI, n (%):<br>Thinness/underweight: 1 (4)<br>Normal weight: 12 (50)<br>Overweight: 2 (8)<br>Obesity: 1 (4)<br>Missing: 8 (34)<br><u>Adults</u><br>BMI, median (IQR), kg/m^2: 22.3 (20.4-25.0)<br>BMI, n (%):<br>Thinness/underweight: 2 (4)<br>Normal weight: 25 (50)<br>Overweight: 8 (16)<br>Obesity: 2 (4)<br>Missing: 13 (26)

First author, Study name	Year	Country	Basis for PCD diagnosis	Study participants (n)	Age *	Sex (% female)	Parameter assessment	Growth comparator	Findings (overall and per subgroups, when available)
Asseri [29]	2023	SA	Two out of four of the following criteria: (a) a persistent, wet cough that starts in the first 6 months of life, (b) persistent, nasal congestion that starts in the first 6 months of life, (c) the presence of organ laterality abnormalities, and (d) unexplained NRD in infants born at term gestation. Diagnosis of PCD was confirmed by finding two mutations in one or two genes that are known to cause PCD	28	7.5 (3-13), median (IQR)	50	NA	NA	Height, median (IQR), cm: 115 (78-128)<br>Weight, median (IQR), kg: 18 (8-25)<br>BMI, median (IQR), kg/m^2: 15 (12-16)<br>BMI, median (IQR), percentile: 12 (3.5-46)<br>BMI percentile < 5%, n (%): 8 (28.6)
Cohen-Cymberknoh [30]	2023	IS	ATS guidelines	15	Children: 13 (11-15.5), median (IQR); Adults: 27 (27-37), median (IQR)	47	Collected via electronic medical records	NA	Children<br>BMI, median (IQR), percentile: 53 (3-87)<br>Adults<br>BMI, median (IQR), kg/m^2: 27.9 (23.1-31.1)

First author, Study name	Year	Country	Basis for PCD diagnosis	Study participants (n)	Age *	Sex (% female)	Parameter assessment	Growth comparator	Findings (overall and per subgroups, when available)
Fein [31]	2023	DE	ERS guidelines	44	Age at first appointment: 9.92 ± 5.74 [0.14-24.13], mean ± SD [range]	50	Recorded during visits	NA	BMI, mean, during the observation period: 19.99 **
Gatt [32]	2023	CA	ATS guidelines	28	Age at admission: 14.8 (10.9-16.7), median (IQR)	57	NA	NA	Patients achieving ≥90% of baseline FEV1pp values at the end of admission (responders)<br>BMI, median (IQR), z-score: -0.4 (-1.7-0.3)<br>Nonresponders<br>BMI, median (IQR), z-score: -2.4 (-4.0 - -0.9)
Graziano [33]	2023	IT	Genetically and/or by electron microscopy	21	17.6 ± 6.0, mean ± SD	43	Chart review	NA	BMI, mean ± SD, kg/m^2: 21.5 ± 3.0
Kinghorn, GDMCC [34]	2023	International	Abnormal ciliary ultrastructure by TEM and/or identification of two pathogenic variants in a PCD-associated gene together with compatible clinical features	141	Age at CT scan: 8.5 ± 4.6 [0.4-18.5], mean ± SD [range]	50	NA	CDC data	Total study population<br>Height, mean ± SD, percentile: 44.5 ± 29.4<br>Weight, mean ± SD, percentile: 49.5 ± 31.8<br>BMI, mean ± SD, percentile: 54.2 ± 30.8<br>Participants with ODA<br>Height, mean ± SD, percentile: 50.1 ± 30.6<br>Weight, mean ± SD, percentile: 59.0 ± 32.9<br>BMI, mean ± SD, percentile: 61.3 ± 29.8

First author, Study name	Year	Country	Basis for PCD diagnosis	Study participants (n)	Age *	Sex (% female)	Parameter assessment	Growth comparator	Findings (overall and per subgroups, when available)
Kinghorn, GDMCC [34] (cont.)									Participants with ODA/IDA<br>Height, mean ± SD, percentile: 46.3 ± 30.1<br>Weight, mean ± SD, percentile: 48.0 ± 34.7<br>BMI, mean ± SD, percentile: 49.3 ± 35.1<br>Participants with IDA/MTD<br>Height, mean ± SD, percentile: 38.5 ± 24.8<br>Weight, mean ± SD, percentile: 37.0 ± 24.0<br>BMI, mean ± SD, percentile: 45.1 ± 26.0<br>Participants with Normal/Near Normal<br>Height, mean ± SD, percentile: 39.8 ± 32.3<br>Weight, mean ± SD, percentile: 48.9 ± 32.2<br>BMI, mean ± SD, percentile: 54.6 ± 34.4
Rumman [35]	2023	PS	Diagnostic TEM and/or genetic testing, nNO, highly likely clinical picture	68, 28 for our parameter of interest	Age at diagnosis: 10.0 [3 mon-40], mean [range]	40	Recorded by clinician	WHO data	BMI, median [range], z-score: -0.36 [-3.03-2.57]
Zhou [36]	2023	CN	ERS guidelines as described in a previous publication [51]. In that publication, it is stated “A total of 72 children, suspected of PCD, were	72	11.3 [6.7-22.8)], median [range]	53	NA	WHO data	Discrepant information given in the manuscript.<br>Either BMI, mean [range], z-score: -1.35 [-0.41-0.40], or<br>BMI, mean [range], z-score: 1.35 [-0.41-0.40]

First author, Study name	Year	Country	Basis for PCD diagnosis	Study participants (n)	Age *	Sex (% female)	Parameter assessment	Growth comparator	Findings (overall and per subgroups, when available)
Zhou [36] (cont.)			invited, of these, 18 children no longer fulfilled inclusion criteria, and 4 were “inconclusive”						
Ademhan Tural [37]	2024	TR	Clinical signs and symptoms consistent with the disease, a low nNO as well as evidence for structural ciliary defects on electron microscopy of a nasal or bronchial brush sample and/or two known disease-causing mutations on genetic testing	14	12.5 (10.8-19.5), median (Q1-Q3)	71	Retrieved from the patients' records	WHO data	BMI, median (Q1-Q3), kg/m^2: 19.5 (14.9-21.0)<br>BMI, median (Q1-Q3), z-score: -0.57 (-1.5-0.64)<br>BMI, median (Q1-Q3), percentile: 23.8 (9.9-58.2)
Ewen, PROGNOSIS [38]	2024	DE	Patients’ history and etiologic testing data (nNO, HSVM, TEM, genetic testing, presence of Kartagener syndrome [situs inversus], or historical saccharin test)	87	42 ± 15, mean ± SD	66	NA	NA	BMI, mean ± SD, kg/m^2:<br>22.8 ± 3.7<br>BMI, categories, n (%):<br><18.5: 7 (8.1)<br>18.5-30: 75 (86.2)<br>>30: 5 (5.8)

First author, Study name	Year	Country	Basis for PCD diagnosis	Study participants (n)	Age *	Sex (% female)	Parameter assessment	Growth comparator	Findings (overall and per subgroups, when available)
Kaspy, GDMCC [39]	2024	International	Abnormal ciliary ultrastructure on TEM, trait-specific pathogenic or likely pathogenic variants in a single PCD gene, or both	397	SA: 7.5 [2.4-12.3], mean [range]<br>No SA: 8.2 [3.8-12.6], mean [range]	52	NA	NA	<u>SA</u><br>BMI, mean ± SD, z-score: -0.3 ± 1.7<br><u>No SA</u><br>BMI, mean ± SD, z-score: 0.1 ± 1.4
Kinghorn, GDMCC [40]	2024	International	Abnormal ciliary ultrastructure by TEM and/or identification of two pathogenic variants in a PCD-associated gene, together with compatible clinical features	136	8.4 ± 4.6, mean ± SD	51	Collected at annual study visits	CDC data	<u>Total study population</u><br>Height, estimated mean (95% CI), percentile, age 2: 46.8 (39.4-54.2)<br>Height, estimated mean (95% CI), percentile, age 6: 45.9 (41.7-50.1)<br>Height, estimated mean (95% CI), percentile, age 10: 45.1 (41.2-49.0)<br>Height, estimated mean (95% CI), percentile, age 14: 47.0 (43.1-50.9)<br>Weight, estimated mean (95% CI), percentile, age 2: 50.6 (44.0-57.2)<br>Weight, estimated mean (95% CI), percentile, age 6: 50.9 (47.2-54.7)<br>Weight, estimated mean (95% CI), percentile, age 10: 51.3 (47.6-54.9)<br>Weight, estimated mean (95% CI), percentile, age 14: 52.8 (49.1-56.4)<br>BMI, estimated mean (95% CI), percentile, age 2: 55.0 (48.1-61.8)<br>BMI, estimated mean (95% CI), percentile, age 6: 55.7 (52.0-59.4)<br>BMI, estimated mean (95% CI), percentile, age 10: 56.3 (52.8-59.8)<br>BMI, estimated mean (95% CI), percentile, age 14: 54.5 (51.0-57.9)<br><u>Participants with IDA/MTD</u>

First author, Study name	Year	Country	Basis for PCD diagnosis	Study participants (n)	Age *	Sex (% female)	Parameter assessment	Growth comparator	Findings (overall and per subgroups, when available)
Kinghorn, GDMCC [40] (cont.)									Height, estimated mean (95% CI), percentile, age 2: 40.5 (30.2-50.8)<br>Height, estimated mean (95% CI), percentile, age 6: 41.5 (35.9-47.1)<br>Height, estimated mean (95% CI), percentile, age 10: 42.5 (36.2-48.8)<br>Height, estimated mean (95% CI), percentile, age 14: 43.9 (37.1-50.8)<br>Weight, estimated mean (95% CI), percentile, age 2: 42.6 (34.0-51.2)<br>Weight, estimated mean (95% CI), percentile, age 6: 41.3 (36.3-46.3)<br>Weight, estimated mean (95% CI), percentile, age 10: 40.0 (34.1-46.0)<br>Weight, estimated mean (95% CI), percentile, age 14: 38.0 (31.4-44.5)<br>BMI, estimated mean (95% CI), percentile, age 2: 47.9 (38.4-57.4)<br>BMI, estimated mean (95% CI), percentile, age 6: 47.5 (42.5-52.5)<br>BMI, estimated mean (95% CI), percentile, age 10: 46.8 (41.1-52.5)<br>BMI, estimated mean (95% CI), percentile, age 14: 39.8 (33.6-46.0)<br><u>Participants classified as PCD-Other</u><br>Height, estimated mean (95% CI), percentile, age 2: 51.8 (42.4-61.3)<br>Height, estimated mean (95% CI), percentile, age 6: 49.0 (43.8-54.1)<br>Height, estimated mean (95% CI), percentile, age 10: 46.3 (41.8-50.7)<br>Height, estimated mean (95% CI), percentile, age 14: 47.7 (43.4-52.0)<br>Weight, estimated mean (95% CI), percentile, age 2: 58.5 (49.9-67.2)

First author, Study name	Year	Country	Basis for PCD diagnosis	Study participants (n)	Age *	Sex (% female)	Parameter assessment	Growth comparator	Findings (overall and per subgroups, when available)
Kinghorn, GDMCC [40] (cont.)									Weight, estimated mean (95% CI), percentile, age 6: 57.2 (52.6-61.9)<br>Weight, estimated mean (95% CI), percentile, age 10: 56.0 (51.9-60.1)<br>Weight, estimated mean (95% CI), percentile, age 14: 57.1 (53.1-61.1)<br>BMI, estimated mean (95% CI), percentile, age 2: 61.4 (52.8-70.1)<br>BMI, estimated mean (95% CI), percentile, age 6: 61.0 (56.5-65.6)<br>BMI, estimated mean (95% CI), percentile, age 10: 60.6 (56.7-64.4)<br>BMI, estimated mean (95% CI), percentile, age 14: 58.6 (54.9-62.4)
Koucký [41]	2024	CZ	Conclusive genetics and/or TEM findings	15	Age at iPFT: 35.9 wks (13.9-74.0), median (IQR)	NA	NA	NA	Length, median (IQR), cm, at iPFT: 71.0 (60.5-80.0)<br>Length, median (IQR), z-score, at iPFT: -0.32 (-0.81-0.47)<br>Weight, median (IQR), g, at birth: 3500 (3210-3840)<br>Weight, median (IQR), kg, at iPFT: 9.2 (5.7-11.0)<br>Weight, median (IQR), z-score, at iPFT: -0.43 (-1.63-0.63)
Lam, EPIC-PCD [42]	2024	International	ERS guidelines; Ultrastructural defects were categorised based on the international consensus guideline for reporting TEM results	457, 379 for our parameters of interest	15 (10-24), median (IQR)	46	Height and weight reported at ENT or spirometry visit date	WHO data	Total study population<br>BMI classification, n (%), classification definition provided:<br>Thinness/underweight: 26 (6)<br>Normal weight: 225 (49)<br>Pre-obesity/overweight: 66 (14)<br>Obese/obesity class I: 24 (5)<br>Obesity class II: 7 (2)<br>Obesity class III: 31 (7)<br>Missing: 78 (17)

First author, Study name	Year	Country	Basis for PCD diagnosis	Study participants (n)	Age *	Sex (% female)	Parameter assessment	Growth comparator	Findings (overall and per subgroups, when available)
Lam, EPIC-PCD [42] (cont.)									Children<br>BMI, median (IQR), z-score, 0-6 years: -0.5 (-1.3-0.4)<br>BMI classification, n (%), classification definition provided, 0-6 years:<br>Thinness/underweight: 1 (2)<br>Normal weight: 6 (13)<br>Pre-obesity/overweight: 1 (2)<br>Obese/obesity class I: 0<br>Obesity class II: 0<br>Obesity class III: 0<br>Missing: 39 (83)<br>BMI, median (IQR), z-score, 7-14 years: -0.06 (-0.9-1.2)<br>BMI classification, n (%), classification definition provided, 7-14 years:<br>Thinness/underweight: 7 (4)<br>Normal weight: 83 (56)<br>Pre-obesity/overweight: 22 (15)<br>Obese/obesity class I: 9 (6)<br>Obesity class II: 0<br>Obesity class III: 0<br>Missing: 28 (19)<br>Adults<br>BMI, median (IQR), z-score, 15-30 years: 0.1 (-0.9-1.0)<br>BMI, mean (IQR), kg/m^2, 15-30 years: 21.1 (19.7-24.1)<br>BMI classification, n (%), classification definition provided, 15-30 years:<br>Thinness/underweight: 12 (7)<br>Normal weight: 107 (62)<br>Pre-obesity/overweight: 25 (14)<br>Obese/obesity class I: 8 (5)<br>Obesity class II: 3 (2)

First author, Study name	Year	Country	Basis for PCD diagnosis	Study participants (n)	Age *	Sex (% female)	Parameter assessment	Growth comparator	Findings (overall and per subgroups, when available)
Lam, EPIC-PCD [42] (cont.)									Obesity class III: 7 (4)<br>Missing: 11 (6)<br>BMI, mean (IQR), kg/m^2, 31-50 years: 23.2 (20.8-27.4)<br>BMI classification, n (%), classification definition provided, 31-50 years:<br>Thinness/underweight: 5 (9)<br>Normal weight: 23 (43)<br>Pre-obesity/overweight: 13 (24)<br>Obese/obesity class I: 3 (6)<br>Obesity class II: 0<br>Obesity class III: 10 (18)<br>Missing: 0<br>BMI, mean (IQR), kg/m^2, >50 years: 28.1 (21.6-35.3)<br>BMI classification, n (%), classification definition provided, >50 years:<br>Thinness/underweight: 1 (3)<br>Normal weight: 6 (17)<br>Pre-obesity/overweight: 5 (15)<br>Obese/obesity class I: 4 (12)<br>Obesity class II: 4 (12)<br>Obesity class III: 14 (41)<br>Missing: 0
Marzook [43]	2024	CA	Two disease-causing variants in a known PCD gene, and/or a classic ultrastructural ciliary defect on electron microscopy, or repeated low nNO values with a	30	Age at study entry: 11.5 (6.2-14.2), median (IQR)	50	NA	NA	BMI, median (IQR), z-score: 0.1 (-0.83-0.54)

First author, Study name	Year	Country	Basis for PCD diagnosis	Study participants (n)	Age *	Sex (% female)	Parameter assessment	Growth comparator	Findings (overall and per subgroups, when available)
Marzook [43] (cont.)			compatible PCD clinical phenotype						
Raidt, ERN-LUNG international PCD Registry [44]	2024	International	Genetically confirmed	1236, 1093 for our parameters of interest	21.6 (15.4-32.2), median (IQR)	50	BMI was calculated from height and weight at clinical appointments	WHO	<u>Total study population</u><br>BMI, median (IQR), kg/m^2: 20.3 (17.4-23.8)<br><u>Individuals <19</u><br>BMI, median (IQR), z-score: 0.00 (-0.7-0.07)<br><u>Individuals >19</u><br>BMI, median (IQR), kg/m^2: 22.7 (20.4-25.6)
Rubbo, PROVALF-PCD [45]	2024	International	Confirmed (i.e. bi-allelic causative mutations in PCD-related genes or hallmark defects on TEM), highly likely or inconclusive (pending additional confirmatory tests) according to the ERS diagnostic guidelines	408	15 (11- 20), median (IQR)	51	NA	NA	<u>Total study population</u><br>BMI, median (IQR), z-score: 0.1 (-0.6-1.0)<br><u>Children</u><br>BMI, median (IQR), z-score: 0.04 (-0.7-0.8)<br><u>Adults</u><br>BMI, median (IQR), z-score: 0.2 (-0.5-1.3)
Holgersen [46]	2025	DK	Confirmed PCD genotype	127, 107 for our parameter of interest	Age at inclusion: 9 (6-17), median (IQR)	50	NA	WHO data	<u>Children</u><br>BMI, median (IQR), z-score, overall: -0.2 (-0.7-0.7)<br>BMI, median (IQR), z-score, C2D/normal: 0.0 (-0.7-0.7)<br>BMI, median (IQR), z-score, MTD/IDA: -0.5 (-0.9 - -0.2)<br>BMI, median (IQR), z-score, ODA: 0.2 (-0.6-1.0)

First author, Study name	Year	Country	Basis for PCD diagnosis	Study participants (n)	Age *	Sex (% female)	Parameter assessment	Growth comparator	Findings (overall and per subgroups, when available)
Holgersen [46] (cont.)									BMI, median (IQR), z-score, ODA/IDA: 0.1 (-0.8-0.8)<br>Adults<br>BMI, median (IQR), kg/m^2, overall: 22.4 (20.9-24.6)<br>BMI, median (IQR), kg/m^2, C2D/normal: 21.9 (20.4-24.1)<br>BMI, median (IQR), kg/m^2, MTD/IDA: 22.1 (21.2-22.4)<br>BMI, median (IQR), kg/m^2, ODA: 23.8 (21.2-25.0)<br>BMI, median (IQR), kg/m^2, ODA/IDA: 24.7 (24.7-24.7)
Kahraman [47]	2025	TR	Descriptive criteria of the guidelines by evaluating anamnesis, family history, physical examination, as well as laboratory, radiological, and/or genetic results. The diagnosis of SIT was performed by chest X-ray, computed tomography, and echocardiography.	48	13 (6.5-16), median (25-75)	46	Recorded, NOS	NA	BMI, median (25-75), kg/m^2: 16.00 (14.73-19.06)<br>BMI classification, n (%), no classification definition provided:<br>Underweight: 24 (50.00)<br>Normal: 20 (41.67)<br>Overweight: 4 (8.33),
Macatangay [48]	2025	US	NA	7	10.2 (3.5-17.7), median (IQR)	57	NA	NA	Height, mean ± SD, cm: 129.9 ± 32.3<br>Weight, mean ± SD, kg: 34.7 ± 21.9<br>BMI, median (IQR), kg/m^2: 16.7 (16.2-24.2)

First author, Study name	Year	Country	Basis for PCD diagnosis	Study participants (n)	Age *	Sex (% female)	Parameter assessment	Growth comparator	Findings (overall and per subgroups, when available)
Mutlu [49]	2025	TR	Results consistent with PCD diagnosis in genetic or HSVM according to ATS and ERS guidelines	27	14.11 ± 3.24, mean ± SD	48	Height, weight, and BMI z-scores were recorded from the patient's medical records	WHO	Height, mean ± SD, z-score: -0.40 ± 1.37<br>Weight, mean ± SD, z-score: -0.64 ± 1.6<br>Discrepant information given in the manuscript. BMI was reported as mean ± SD, but the reporting looks like median (IQR).<br>BMI z-score: -0.28 (-1.44 to 0.77)<br>Malnutrition (BMI z-score ≤ -2), n (%): 3 (11.1%)
Sunman [50]	2025	TR	Cilia video microscopy and/or genetic analysis together with a suggestive clinical history	21	4 (3-5), median (IQR)	44	Device was calibrated daily, and patient details such as sex, height, and weight were recorded	NA	Height, median (IQR), cm: 109 (99.5-119)<br>Weight, median (IQR), kg: 18 (14.5-22)<br>BMI, median (IQR), kg/m^2: 15 (14-16.5)

*Current age is reported, unless otherwise specified. Age reported in years, unless otherwise specified; ** Mean BMI calculated by the review authors' based on information provided in the supplementary material of the publication

Abbreviations: American Thoracic Society (ATS); BMI standard deviation scores (BMI-SDS); Body Mass Index (BMI); Central apparatus (CA); Class 2 defect (C2D); Centers for Disease Control and Prevention (CDC); Confidence interval (CI); Ear-nose-throat (ENT); ENT Prospective International Cohort of Patients with PCD (EPIC-PCD); European Respiratory Society (ERS); Genetic Disorders of Mucociliary Clearance Consortium (GDMCC); High speed video microscopy (HSVM); Infant pulmonary function testing (iPFT); Inner dynein arm (IDA); International PCD cohort (iPCD); Interquartile range (IQR); Microtubular defect (MTD); month (mon); Nasal nitric oxide (nNO); Not available (NA); Not otherwise specified (NOS); Outer dynein arm (ODA); Outer dynein arm with or without inner dynein arm (ODA ± IDA); Primary Ciliary Dyskinesia (PCD); Primary Ciliary Dyskinesia Rule score (PICADAR); Prospective German Non-CF-Bronchiectasis Registry (PROGNOSIS); Prospective Observational Multicentre Study on Variability of Lung Function in Stable PCD patients (PROVALF-PCD); Situs ambiguous (SA); Situs inversus (SI); Standard deviation (SD); Standard deviation scores (SDS); Swiss PCD registry (CH-PCD); Transmission electron microscopy (TEM); weeks (wks)

Country-related abbreviations: Canada (CA); China (CN); Cyprus (CY); Czechia (CZ); Denmark (DK); France (FR); Germany (DE); Israel (IS); Italy (IT); Netherlands (NL); Palestine (PS); Portugal (PT); Saudi Arabia (SA); Switzerland (CH); Türkiye (TR); United Kingdom (UK); United States (US)

References

1 Wells GD, Wilkes DL, Schneiderman JE, *et al.* Skeletal muscle metabolism in cystic fibrosis and primary ciliary dyskinesia. *Pediatr Res* 2011; 69: 40–45.

2 Valerio G, Giallauria F, Montella S, *et al.* Cardiopulmonary assessment in primary ciliary dyskinesia. *Eur J Clin Invest* 2012; 42: 617–622.

3 Fowler CJ, Olivier KN, Leung JM, *et al.* Abnormal nasal nitric oxide production, ciliary beat frequency, and toll-like receptor response in pulmonary nontuberculous mycobacterial disease epithelium. *Am J Respir Crit Care Med* 2013; 187: 1374–1381.

4 Svobodová T, Djakow J, Zemková D, *et al.* Impaired Growth during Childhood in Patients with Primary Ciliary Dyskinesia. *Int J Endocrinol* 2013; 2013: 731423.

5 Cohen-Cymberknoh M, Simanovsky N, Hiller N, *et al.* Differences in disease expression between primary ciliary dyskinesia and cystic fibrosis with and without pancreatic insufficiency. *Chest* 2014; 145: 738–744.

6 Maglione M, Bush A, Nielsen KG, *et al.* Multicenter analysis of body mass index, lung function, and Sputum microbiology in primary ciliary dyskinesia. *Pediatr Pulmonol* 2014; 49: 1243–1250.

7 Mirra V, Caffarelli C, Maglione M, *et al.* Hypovitaminosis D: a novel finding in primary ciliary dyskinesia. *Ital J Pediatr* 2015; 41: 14.

8 Yiallouros PK, Kouis P, Middleton N, *et al.* Clinical features of primary ciliary dyskinesia in Cyprus with emphasis on lobectomized patients. *Respir Med* 2015; 109: 347–356.

9 Cohen-Cymberknoh M, Weigert N, Gileles-Hillel A, *et al.* Clinical impact of Pseudomonas aeruginosa colonization in patients with Primary Ciliary Dyskinesia. *Respir Med* 2017; 131: 241–246.

10 Frija-Masson J, Bassinet L, Honoré I, *et al.* Clinical characteristics, functional respiratory decline and follow-up in adult patients with primary ciliary dyskinesia. *Thorax* 2017; 72: 154–160.

11 Goutaki M, Halbeisen FS, Spycher BD, *et al.* Growth and nutritional status, and their association with lung function: a study from the international Primary Ciliary Dyskinesia Cohort. *Eur Respir J* 2017; 50: 12.

12 Irving S, Dixon M, Fassad MR, *et al.* Primary Ciliary Dyskinesia Due to Microtubular Defects is Associated with Worse Lung Clearance Index. *Lung* 2018; 196: 231–238.

13 Marino LV, Harris A, Johnstone C, *et al.* Characterising the nutritional status of children with primary ciliary dyskinesia. *Clin Nutr* 2019; 38: 2127–2135.

14 Emiralioğlu N, Taşkıran EZ, Koşukcu C, *et al.* Genotype and phenotype evaluation of patients with primary ciliary dyskinesia: First results from Turkey. *Pediatr Pulmonol* 2020; 55: 383–393.

15 Piatti G, De Santi MM, Farolfi A, *et al.* Exacerbations and Pseudomonas aeruginosa colonization are associated with altered lung structure and function in primary ciliary dyskinesia. *BMC Pediatr* 2020; 20: 158.

16 Pifferi M, Bush A, Mariani F, *et al.* Lung Function Longitudinal Study by Phenotype and Genotype in Primary Ciliary Dyskinesia. *Chest* 2020; 158: 117–120.

17 Rubbo B, Best S, Hirst RA, *et al.* Clinical features and management of children with primary ciliary dyskinesia in England. *Arch Dis Child* 2020; 105: 724–729.

18 Alzaid M, Al-Mobaireek K, Almannai M, *et al.* Clinical and molecular characteristics of primary ciliary dyskinesia: A tertiary care centre experience. *Int J Pediatr Adolesc Med* 2021; 8: 258–263.

19 Constant C, Descalço A, Silva AM, *et al.* Implementing nitrogen multiple breath washout as a clinical tool – A feasibility study. *Pulmonology* 2021; 27: 569–571.

20 Kilinc AA, Cebi MN, Ocak Z, *et al.* The Relationship between Genotype and Phenotype in Primary Ciliary Dyskinesia Patients. *Sisli Etfal Hastan Tp Bul* 2021; 55: 188–192.

21 King L, White H, Clifton I, *et al.* Nutritional status and intake in patients with non-cystic fibrosis bronchiectasis (NCFB) - a cross sectional study. *Clin Nutr* 2021; 40: 5162–5168.

22 Pifferi M, Bush A, Mulé G, *et al.* Longitudinal lung volume changes by ultrastructure and genotype in primary ciliary dyskinesia. *Ann Am Thorac Soc* 2021; 18: 963–970.

23 Verkleij M, Appelman I, Altenburg J, *et al.* Anxiety and depression in Dutch patients with primary ciliary dyskinesia and their caregivers: associations with health-related quality of life. *ERJ Open Res* 2021; 7.

24 Firat M, Bosnak-Guclu M, Sismanlar-Eyuboglu T, *et al.* Respiratory muscle strength, exercise capacity and physical activity in patients with primary ciliary dyskinesia: A cross-sectional study. *Respir Med* 2022; 191: 106719.

25 Guan Y, Zhang X, Yang H, *et al.* Long-Term Azithromycin Treatment in Pediatric Primary Ciliary Dyskinesia: A Retrospective Study. *Front Pediatr* 2022; 10: 905253.

26 Halbeisen FS, Pedersen ESL, Goutaki M, *et al.* Lung function from school age to adulthood in primary ciliary dyskinesia. *Eur Respir J* 2022; 60: 10.

27 Kos R, Israëls J, van Gogh CDL, *et al.* Primary ciliary dyskinesia in Volendam: Diagnostic and phenotypic features in patients with a CCDC114 mutation. *Am J Med Genet C Semin Med Genet* 2022; 190: 89–101.
28 Lam YT, Pedersen ESL, Schreck LD, *et al.* Physical activity, respiratory physiotherapy practices, and nutrition among people with primary ciliary dyskinesia in Switzerland - a cross-sectional survey. *Swiss Med Wkly* 2022; 152: w30221.
29 Asseri AA, Shati AA, Asiri IA, *et al.* Clinical and Genetic Characterization of Patients with Primary Ciliary Dyskinesia in Southwest Saudi Arabia: A Cross Sectional Study. *Children* 2023; 10.
30 Cohen-Cymberknoh M, Lehavi M, Gileles-Hillel A, *et al.* Changes in Sleep in Children and Adults with Cystic Fibrosis and Primary Ciliary Dyskinesia over Time and after CFTR Modulator Therapy. *J Clin Med* 2023; 12.
31 Fein V, Maier C, Schlegtendal A, *et al.* Risk factors for the deterioration of pulmonary function in primary ciliary dyskinesia. *Pediatr Pulmonol* 2023; 58: 1950–1958.
32 Gatt D, Shaw M, Waters V, *et al.* Treatment response to pulmonary exacerbation in primary ciliary dyskinesia. *Pediatr Pulmonol* 2023; 58: 2857–2864.
33 Graziano S, Ullmann N, Rusciano R, *et al.* Comparison of mental health in individuals with primary ciliary dyskinesia, cystic fibrosis, and parent caregivers. *Respir Med* 2023; 207.
34 Kinghorn B, Rosenfeld M, Sullivan E, *et al.* Airway disease in children with primary ciliary dyskinesia: impact of ciliary ultrastructure defect and genotype. *Ann Am Thorac Soc* American Thoracic Society; 2023; 20: 539–547.
35 Rumman N, Fassad MR, Driessens C, *et al.* The Palestinian primary ciliary dyskinesia population: first results of the diagnostic and genetic spectrum. *ERJ Open Res* 2023; 9.
36 Zhou W, Guo Z, Chen J, *et al.* Airway microbiota correlated with pulmonary exacerbation in primary ciliary dyskinesia patients. *Microbiol Spectr* 2023; 11.
37 Ademhan Tural D, Kasikci M, Eryilmaz Polat S, *et al.* The airway microbiota in siblings with primary ciliary dyskinesia: Related factors and correlation with clinical characteristics. *Pediatr Pulmonol* Wiley Online Library; 2024; 59: 695–706.
38 Ewen R, Pink I, Sutharsan S, *et al.* Primary Ciliary Dyskinesia in Adult Bronchiectasis: Data from the German Bronchiectasis Registry PROGNOSIS. *Chest* 2024; 166: 938–950.
39 Kaspy KR, Dell SD, Davis SD, *et al.* Situs Ambiguus Is Associated With Adverse Clinical Outcomes in Children With Primary Ciliary Dyskinesia. *Chest* 2024; 165: 1070–1081.
40 Kinghorn B, Rosenfeld M, Sullivan E, *et al.* Comparison of Longitudinal Outcomes in Children with Primary Ciliary Dyskinesia and Cystic Fibrosis. *Ann Am Thorac Soc* 2024; 21: 1723–1732.
41 Koucký V, Martinů V, Koucký M. Impaired lung function in infants with primary ciliary dyskinesia: A pilot Czech study. *Pediatr Pulmonol* 2024; 59: 1124–1127.
42 Lam YT, Papon JF, Alexandru M, *et al.* Association between upper and lower respiratory disease among patients with primary ciliary dyskinesia: an international study. *ERJ Open Res* 2024; 10.
43 Marzook N, Dubrovsky AS, Muchantef K, *et al.* Lung ultrasound in children with primary ciliary dyskinesia or cystic fibrosis. *Pediatr Pulmonol* 2024; 59: 3391–3399.
44 Raidt J, Riepenhausen S, Pennekamp P, *et al.* Analyses of 1236 genotyped primary ciliary dyskinesia individuals identify regional clusters of distinct DNA variants and significant genotype–phenotype correlations. *Eur Respir J* 2024; 64.
45 Rubbo B, Kant A, Zhang K, *et al.* Associations between respiratory pathogens and lung function in primary ciliary dyskinesia: cross-sectional analysis from the PROVALF-PCD cohort. *ERJ Open Res* 2024; 10.
46 Holgersen MG, Marthin JK, Raidt J, *et al.* Long-Term Lung Function and Pseudomonas aeruginosa Infection in Genotyped Primary Ciliary Dyskinesia. *Ann Am Thorac Soc* 2025; 22: 216–225.
47 Kahraman FU, Jafarov U, Yazan H, *et al.* Evaluation of the Clinical and Genetic Characteristics of Primary Ciliary Dyskinesia Patients With Situs Inversus Totalis. *Birth Defects Res* 2025; 117.
48 Macatangay NJ, Engdahl S, Pettit RS. Tobramycin Pharmacokinetics in Pediatric Patients With Primary Ciliary Dyskinesia. *Pediatr Pulmonol* 2025; 60: e71018.
49 Mutlu Ş, Boşnak Güçlü M, Şişmanlar Eyüboğlu T, *et al.* Upper Extremity Exercise Capacity and Muscle Oxygenation in Patients With Primary Ciliary Dyskinesia. *Pediatr Pulmonol* 2025; 60.
50 Sunman B, Yalcin E, Alboga D, *et al.* Impulse Oscillometry is Useful in Detecting Lung Function Abnormalities in Preschoolers with Primary Ciliary Dyskinesia but Not Cystic Fibrosis: A Cross-Sectional Study Results. *Pediatr Allergy Immunol Pulmonol* 2025; 38: 71–77.
51 Guo Z, Chen W, Wang L, *et al.* Clinical and Genetic Spectrum of Children with Primary Ciliary Dyskinesia in China. *J Pediatr* 2020; 225: 157-165.e5.
